# Thymic epithelial organoids mediate T-cell development

**DOI:** 10.1242/dev.202853

**Published:** 2024-09-03

**Authors:** Tania Hübscher, L. Francisco Lorenzo-Martín, Thomas Barthlott, Lucie Tillard, Jakob J. Langer, Paul Rouse, C. Clare Blackburn, Georg Holländer, Matthias P. Lutolf

**Affiliations:** ^1^ Laboratory of Stem Cell Bioengineering, Institute of Bioengineering, School of Life Sciences and School of Engineering, École Polytechnique Fédérale de Lausanne (EPFL), 1015 Lausanne, Switzerland; ^2^ Pediatric Immunology, Department of Biomedicine, University of Basel, 4058 Basel, Switzerland; ^3^ Centre for Regenerative Medicine, Institute for Regeneration and Repair, School of Biological Sciences, University of Edinburgh, Edinburgh, EH16 4UU, UK; ^4^ Department of Paediatrics, University of Oxford, Oxford, OX3 9DU, UK; ^5^ Institute of Developmental and Regenerative Medicine, University of Oxford, Oxford, OX3 7TY, UK; ^6^ Department of Biosystems Science and Engineering, Eidgenössische Technische Hochschule Zürich (ETHZ), 4056 Basel, Switzerland; ^7^ Institute of Human Biology (IHB), Pharma Research and Early Development, Roche Innovation Center Basel, 4058 Basel, Switzerland

**Keywords:** Thymus, Organoids, Thymic epithelial cells, Thymopoiesis, Mouse, T cells

## Abstract

Although the advent of organoids has opened unprecedented perspectives for basic and translational research, immune system-related organoids remain largely underdeveloped. Here, we established organoids from the thymus, the lymphoid organ responsible for T-cell development. We identified conditions enabling mouse thymic epithelial progenitor cell proliferation and development into organoids with diverse cell populations and transcriptional profiles resembling *in vivo* thymic epithelial cells (TECs) more closely than traditional TEC cultures. In contrast to these two-dimensional cultures, thymic epithelial organoids maintained thymus functionality *in vitro* and mediated physiological T-cell development upon reaggregation with T-cell progenitors. The reaggregates showed *in vivo*-like epithelial diversity and the ability to attract T-cell progenitors. Thymic epithelial organoids are the first organoids originating from the stromal compartment of a lymphoid organ. They provide new opportunities to study TEC biology and T-cell development *in vitro*, paving the way for future thymic regeneration strategies in ageing or acute injuries.

## Teaser

**Highlighted Article:** Establishment of organoids derived from the epithelial cells of the mouse thymus that resemble their *in vivo* counterpart and have thymopoietic ability in reaggregate culture.

## INTRODUCTION

Over the past two decades, organoids have revolutionized the field of stem cell biology. Recapitulating key elements of the architecture, multicellularity or function of their native organs on a smaller scale (Rossi et al., 2018), organoids have opened up unprecedented opportunities for personalized medicine. These three-dimensional (3D) structures derived from stem or progenitor cells have been established from a wide variety of organs, particularly of the endodermal lineage (Rossi et al., 2018). However, despite the availability of organotypic cultures [e.g. tissue explants (Anderson and Jenkinson, 1998; Owen and Ritter, 1969) and reaggregates (Giger et al., 2022; Jenkinson et al., 1992; Wagar et al., 2021)] and engineering methods (Kim et al., 2019) [e.g. scaffolds (Asnaghi et al., 2021; Bourgine et al., 2018; Campinoti et al., 2020; Fan et al., 2015; Hun et al., 2016; Poznansky et al., 2000; Purwada and Singh, 2017) and organ-on-a-chip (Goyal et al., 2022)], bona fide immune system-related organoids are considerably underdeveloped. Modelling lymphoid organs is indeed particularly challenging, largely owing to the intricate crosstalk between the immune cells and stromal cells required for organ development and function (Anderson and Jenkinson, 1998).

One essential organ for adaptive immunity is the thymus as it functions as the site of T-cell development (thymopoiesis). In the thymus, T-cell progenitors undergo lineage commitment and various selection processes to ensure the formation of a diverse, functional and self-tolerant T-cell repertoire, essential for effective immune protection. The instruction of the developing T cells (termed thymocytes) is mostly mediated by thymic epithelial cells (TECs). These stromal cells originate from the pharyngeal endoderm and can be subdivided into cortical and medullary lineages, which mediate successive stages of T-cell development.

The essential thymopoietic ability of TECs is, however, mostly lost *in vitro*, as traditional two-dimensional (2D) cultures fail to maintain their functionality (Anderson and Jenkinson, 1998; Anderson et al., 1998; Mohtashami and Zúñiga-Pflücker, 2006). Alternative approaches employing OP9 or MS5 cell lines have been developed to circumvent this limitation and study T-cell development *in vitro* (Montel-Hagen et al., 2020; Seet et al., 2017), but the absence of TECs still prevents physiological modelling of T-cell selection processes. Other efforts focused on obtaining TECs from pluripotent stem cells (Lai and Jin, 2009; Parent et al., 2013; Ramos et al., 2022; Sun et al., 2013) or through direct reprogramming (Bredenkamp et al., 2014), but these cells largely rely on *in vivo* grafting to reveal thymopoietic functionality. It has also been shown that TECs can form colonies in Matrigel, but these cultures still require feeder cells and their functionality has not been demonstrated (Lepletier et al., 2019; Meireles et al., 2017; Wong et al., 2014). Finally, although TEC functionality is preserved in (reaggregate) thymic organ cultures, these 3D organotypic cultures contain different cell types. Therefore, to date, no conditions allow the expansion of functional TECs independently, highlighting the need for more advanced culture methods. Besides its significance for fundamental research, the possibility of recapitulating thymus function *in vitro*, including TEC-specific selection mechanisms, holds translational potential in terms of thymus regeneration in ageing or acute injuries.

Because organoids have been shown to have improved physiological relevance over standard culture methods for other epithelial cells (Hofer and Lutolf, 2021), we aimed at applying a similar approach to TECs. In light of what has been achieved for other endoderm-derived epithelia, we postulated that TECs could be grown independently of other cell types as 3D organoids in an extracellular matrix-based hydrogel. We identified culture conditions allowing mouse TECs to form organoids mirroring to some extent the native tissue, and proved their functionality through their ability to mediate T-cell development upon reaggregation with T-cell progenitors. This work establishes thymic epithelial organoids with *in vitro* thymopoietic ability. Thymic epithelial organoids are the first demonstration of organoids originating from the stromal compartment of a lymphoid organ.

## RESULTS

### Thymic epithelial cells grow and maintain marker expression in defined organoid culture conditions

**Fig. 1.**
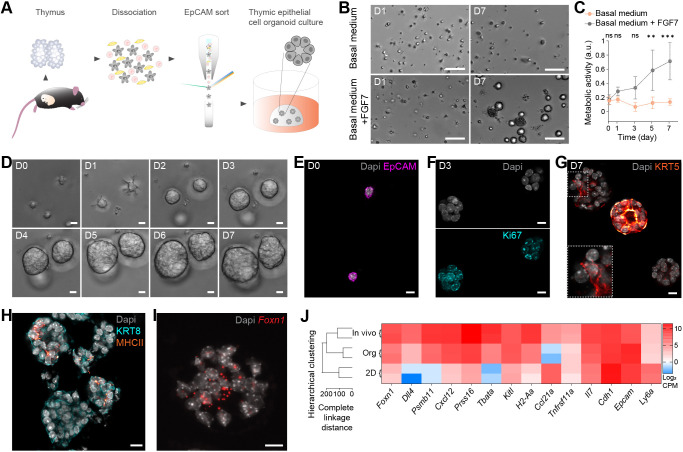
**Thymic epithelial cells grow and maintain marker expression in defined organoid culture conditions.** (A) Workflow to generate thymic epithelial organoids. (B) Brightfield images of thymic epithelial organoids 1 (D1) and 7 (D7) day(s) after seeding, in organoid basal medium supplemented or not with FGF7. (C) Metabolic activity (resazurin assay) of organoids cultured in the same conditions as in B. **P=0.0047, ***P=0.0005; ns, non significant (*P*<0.05) (two-way ANOVA with Šidák multiple comparison test; *n*=15 per condition, from 3 mice). Data represent mean±s.d. a.u., arbitrary units. (D) Brightfield images of sorted TECs from single cells to multicellular organoids over one week. (E-H) Immunofluorescence staining. (E) EpCAM-positive (magenta) TECs immediately after seeding (D0). (F) Proliferative D3 organoids (Ki67; cyan). (G) D7 organoids contain different cell populations, here with medullary cells (KRT5; red) present in various patterns. Inset shows the boxed area at higher magnification. (H) MHCII expression (orange) in D7 organoids co-stained with KRT8 (cyan). Nuclei are stained with DAPI (grey). (I) RNAscope for *Foxn1* in a D7 organoid. Nuclei are counterstained using Spectral DAPI (grey). (J) Gene expression profiling. Left: Dendrogram showing the clustering of thymic epithelial organoids (Org) with freshly extracted TECs (In vivo) and not with TECs cultured in 2D (2D). Right: Heatmap displaying expression of key TEC genes as well as *Cdh1* and *Ly6a* for the same three conditions. *n*=2 mice per condition. All TECs originate from E16.5 thymi. Scale bars: 100 μm (B); 10 μm (D-I).

To establish thymic epithelial organoids, we followed the approach used for other endodermal organs, which included dissociating the tissue, sorting the cells of interest, and seeding them in a basement membrane-rich hydrogel (Matrigel) (Fig. 1A, Fig. S1A). Because organoids mostly develop from stem or progenitor cells, we focused on the embryonic thymus owing to its higher abundance of thymic epithelial progenitor cells compared with the adult organ (Baran-Gale et al., 2020; Kadouri et al., 2020). Although previous attempts to culture TECs often used serum-containing medium (Bonfanti et al., 2010; Campinoti et al., 2020; Wong et al., 2014), we opted for defined organoid basal medium and investigated factors that could promote TEC growth. We hypothesized that mesenchyme-derived factors shown to influence TEC populations (Alawam et al., 2020; Boehm and Swann, 2013; Chaudhry et al., 2016; James et al., 2021) could also be important for TEC growth in organoid cultures. Among these factors, we found FGF7 of particular interest, as it has recently been shown to sustain the expansion of thymic microenvironments without exhausting the epithelial progenitor pools *in vivo* (Nusser et al., 2022). Using embryonic day (E) 16.5 embryonic thymi, we demonstrated that, although sorted TECs failed to grow in organoid basal medium, adding FGF7 to the culture supported organoid formation (Fig. 1B,C). Of note, our culture conditions also permitted organoid growth from neonatal [postnatal day (P) 0] TECs (Fig. S1B), but not from adult TECs.

To monitor organoid development, we performed time-lapse imaging from the time of seeding (Fig. 1D, Movie 1) and found that most organoids were derived from single cells with stem/progenitor properties. Cultures originating from a mixture of single sorted TECs labelled with different CellTrace dyes confirmed the clonal origin of the organoids (Fig. S1C, Movie 2). From total TECs (EpCAM positive), we estimated the organoid forming efficiency as 78±3% (Fig. S1D). Additional experiments in which cortical and medullary TECs (cTECs and mTECs) were sorted independently suggested that both cell types have the potential to form organoids (Fig. S1E).

Immunostaining confirmed that organoids were generated by thymus-derived EpCAM-positive cells (Fig. 1E). Single cells formed small 3D organoids in which a large majority of cells were positive for Ki67 after 3 days (Fig. 1F), and contained both proliferating and non-proliferating cells 4 days later (Fig. S1F). To investigate whether these cell populations could recapitulate TEC diversity, including cortical and medullary types, we stained organoids for the cTEC marker keratin 8 (KRT8), as well as for keratin 5 (KRT5) and with the lectin UEA1 as mTEC markers. Overall, our TEC culture system demonstrated a canonical feature of organoids in the emergence of different cell types, with varying degrees of KRT5 expression (Fig. 1G) and the presence of both KRT8-positive and UEA1-reactive populations (Fig. S1G). In addition, some cells were positive for MHCII (Fig. 1H), an important marker of TEC functionality required for the development of CD4^+^ T cells (Kadouri et al., 2020). Because TEC differentiation, function and maintenance depend on the transcription factor *Foxn1* (Žuklys et al., 2016), we further sought to detect transcripts for this master regulator using RNAscope. Unlike in standard 2D cultures, where it is highly downregulated (Anderson et al., 1998; Mohtashami and Zúñiga-Pflücker, 2006), clear *Foxn1* expression could be observed in organoids (Fig. 1I).

To benchmark thymic epithelial organoids against standard 2D cultures, we performed bulk RNA sequencing. Unsupervised hierarchical clustering showed higher transcriptional similarity of thymic epithelial organoids to freshly extracted TECs (*in vivo*) than to 2D-cultured TECs (Fig. 1J, left). Similarly, differential expression analysis (Table S1) showed that the expression levels of key TEC genes, including *Foxn1*, *Dll4* and *Psmb11*, were more similar between *in vivo* TECs and organoids compared to 2D-cultured TECs (Fig. 1J, right). The expression pattern of *Ccl21a* and *Tnfrsf11a* (encoding a protein also known as RANK), well-known mTEC genes, was, however, not recapitulated in organoids. As previously reported (Anderson et al., 1998), *Il7* expression was maintained, and that of *Cdh1* slightly enhanced in culture in general. Conversely, *Ly6a*, a marker of specific TEC subpopulations (Klein et al., 2023), was highly upregulated in 2D. We also compared *Foxn1* targets (Žuklys et al., 2016) among the three conditions (Fig. S1H). Whereas the genes with the highest expression in freshly extracted TECs were relatively well maintained in organoids, this was not necessarily the case for 2D-cultured TECs. Lastly, gene set enrichment analysis performed on organoids at different time points confirmed the proliferation peak observed with staining (Fig. S1I).

Collectively, these findings show that the defined culture conditions identified herein allow TECs (1) to grow independently of other cell types and (2) to form 3D organoids containing diverse cell populations and that are transcriptionally closer to *in vivo* TECs than to traditional monolayer cultures.

### TECs cultured as organoids show *in vitro* functionality when reaggregated with T-cell progenitors

**Fig. 2.**
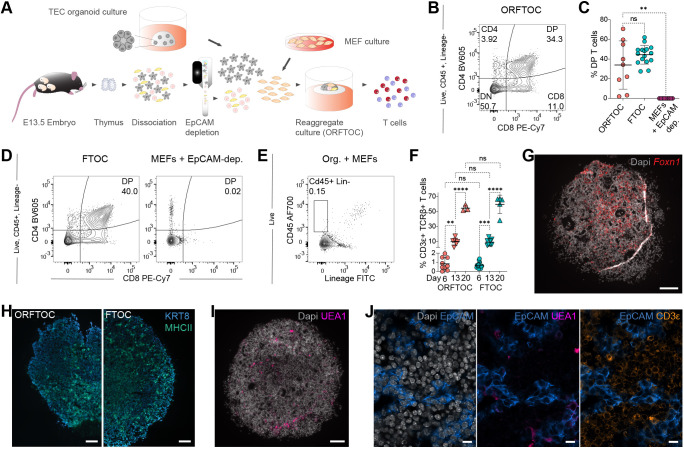
**TECs cultured as organoids show *in vitro* functionality when reaggregated with T-cell progenitors.** (A) Workflow to generate organoid reaggregate fetal thymic organ cultures (ORFTOCs). (B) Flow cytometry plot showing T-cell development in D6 ORFTOCs. (C) Proportion of DP thymocytes at D6 in ORFTOCs and controls [fetal thymic organ cultures (FTOCs) and reaggregates containing only MEFs and the EpCAM-depleted cells from thymic lobes]. **P=0.0047; ns, not significant (*P*>0.05) (Kruskal–Wallis test with Dunn's multiple comparison test; *n*=9, 16 and 9 for ORFTOCs, FTOCs and reaggregates from MEFs+EpCAM-depleted cells, respectively, from 9 independent experiments). (D,E) Flow cytometry plots. (D) T-cell development at D6 in controls (left: FTOCs; right: reaggregates from MEFs+EpCAM-depleted cells). (E) Absence of a CD45– Lineage^−^ (Lin^−^) population in control reaggregates containing only TECs cultured as organoids and MEFs. Gating strategy is indicated on the left for all flow cytometry plots. (F) Proportion of CD3ε-positive, TCRβ-positive cells in ORFTOCs and FTOCs at D6, D13 and D20. **P=0.0021, ***P=0.0003, ****P<0.0001; ns, not significant (*P*>0.05) (two-way ANOVA with Tukey's multiple comparison test; *n*=9, 6, 3, 16, 8 and 5 for D6 ORFTOCs, D13 ORFTOCs, D20 ORFTOCs, D6 FTOCs, D13 FTOCs and D20 FTOCs, respectively, from at least 3 independent experiments). All graphs represent individual datapoints with mean±s.d. (G) RNAscope for *Foxn1* in a D13 ORFTOC. Nuclei are counterstained using Spectral DAPI (grey). (H-J) Immunofluorescence images. (H) KRT8 (blue) and MHCII (green) staining in a D13 ORFTOC (left) and a D13 FTOC (right). (I) Medullary cells (UEA1 reactivity; magenta) in a D13 ORFTOC. (J) Epithelial cells (EpCAM; blue), medullary cells (UEA1 reactivity; bright pink) and T cells (CD3ε; amber) in a D13 ORFTOC. DAPI stains nuclei (grey). ORFTOCs were formed with TECs cultured as organoids originating from E16.5 thymi, with the EpCAM-depleted fraction of cells from E13.5 thymi and with MEFs. FTOCs were from E13.5 thymi. Scale bars: 100 µm (G-I); 10 μm (J).

To test the functionality of thymic epithelial organoids (i.e. their ability to mediate thymopoiesis), we recapitulated the well-known reaggregate fetal thymic organ culture (RFTOC) approach, wherein selected thymic cell populations are reaggregated together and cultured at the air–liquid interface (Jenkinson et al., 1992). To do so, we dissociated TECs cultured as organoids and reaggregated them with an EpCAM-depleted single-cell suspension obtained from E13.5 thymi. We performed EpCAM depletion to keep the mesenchymal cells, which have been proven to be essential for T-cell development (Anderson et al., 1993). We chose E13.5 embryonic thymi as source of T-cell precursors because they contain thymocytes at the earliest stages of development, prior to the expression of CD4 and CD8 (thus referred to as double negative, DN) (Fig. S2A). This facilitates the monitoring of whether T-cell development occurs in the reaggregates. To improve handling, mouse embryonic fibroblasts (MEFs) were added to the reaggregates, as done previously (Sheridan et al., 2009). We termed the RFTOCs formed with TECs from the organoid cultures organoid RFTOCs (ORFTOCs) (Fig. 2A).

After 6 days in culture, ORFTOCs were dissociated and analysed by flow cytometry (Fig. S2B). At this point, thymocytes expressing both CD4 and CD8 (termed double positive, DP) and constituting a developmental stage following the DN phenotype could be readily detected (Fig. 2B,C), indicating that organoid-derived TECs mediated physiological progression of thymocyte maturation. Notably, the proportion of DPs was similar to that observed in cultured intact thymic lobes (i.e. fetal thymic organ cultures, FTOCs) (Fig. 2C,D). Conversely, reaggregating only the EpCAM-depleted fraction of E13.5 thymi and MEFs did not yield DP thymocytes (Fig. 2C,D, Fig. S2C), demonstrating that organoid-derived TECs are necessary for T-cell development in ORFTOCs. Reaggregates with only organoid-derived TECs and MEFs served as negative control and did not produce immune (CD45^+^) cells (Fig. 2E), in contrast to the other conditions (Fig. S2B-D). Lastly, we formed reaggregates containing only organoid-derived TECs and the EpCAM-depleted fraction of E13.5 thymi to exclude the influence of MEFs on thymocyte development (Fig. S2E).

Of note, reaggregates formed with organoids originating from neonatal TECs were also capable of mediating T-cell development (Fig. S2F). We corroborated our findings and avoided the EpCAM-depletion step by reaggregating organoid-derived TECs with the earliest DN subpopulation (DN1) sorted from adult mice and MEFs and could also observe a progression in thymocyte maturation (Fig. S2G). The developmental kinetics was, however, faster in ORFTOCs containing E13.5-derived cells (Fig. 2B), as expected for first wave early T-cell precursors (Rothenberg, 2021).

After 6 days in culture, only a few cells expressed high levels of the αβ–T-cell receptor complex (TCR) (Fig. 2F, Fig. S2H). Extending ORFTOC culture period from 6 to 13, or even 20, days allowed thymocyte maturation to progress further, as shown by the increased proportion and number of TCRβ^+^ cells and their differentiation into CD4^+^ or CD8^+^ single-positive (SP) thymocytes (Fig. 2F, Fig. S2I-L). FTOCs served again as reference and demonstrated a comparable proportion of CD3^+^ TCRβ^+^ (Fig. S2M) and SP (Fig. S2N) cells.

As expected for functional TECs, ORFTOCs were positive for *Foxn1* (Fig. 2G). Morphologically, ORFTOCs also presented similarities to FTOCs, here highlighted by KRT8 and MHCII staining (Fig. 2H). UEA1 reactivity identified medullary cells throughout ORFTOCs (Fig. 2I,J), and CD3ε staining confirmed the presence of T cells in between EpCAM-positive TECs (Fig. 2J).

In summary, we demonstrated that thymic epithelial organoids maintain their functionality and, when reaggregated with T-cell progenitors, mediate thymopoiesis in a similar manner to intact thymic lobe cultures.

### ORFTOCs recapitulate *in vivo*-like TEC and T-cell population diversity and physiological T-cell development

**Fig. 3.**
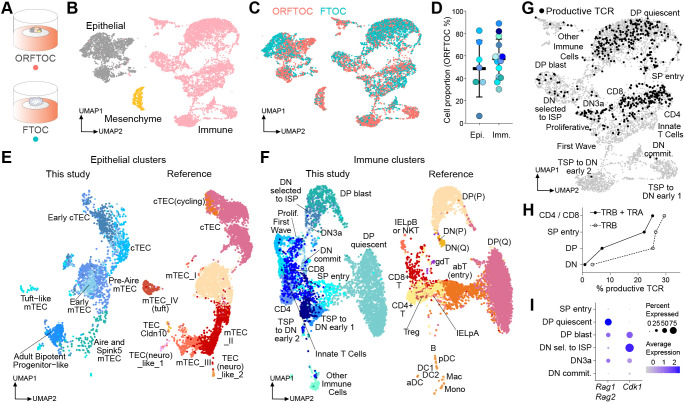
**ORFTOCs recapitulate *in vivo*-like TEC and T-cell population diversity and physiological T-cell development.** (A) D13 ORFTOCs and D13 FTOCs (two pooled for each group) were analysed by scRNAseq with hashtag antibodies. (B) Uniform Manifold Approximation and Projection (UMAP) with three clusters corresponding to the main input populations (epithelial, immune and mesenchymal cells). (C) UMAP for ORFTOC and FTOC cell distribution in the different clusters. (D) ORFTOC proportion for each cluster (circle) within the epithelial or immune main populations. Mean ORFTOC proportion and s.d. are indicated. Circle colours match the cluster colours in E and in Fig. S3A,C (for epithelial and immune cells, respectively). No outliers within epithelial or immune compartments were identified by Grubbs test. (E,F) UMAPs for the integration of the epithelial (E) and immune (F) clusters identified in this study (left) with the mouse datasets of the Park et al. atlas (Park et al., 2020) (right). (G) UMAP of the immune cluster (grey), highlighting cells identified as productive and bearing both TCR chains (black). (H) Proportion of productive cells with rearranged TRB or both TRA and TRB chains for the main thymocyte developmental stages. (I) Average expression level (dot colour) and percentage (dot size) of cells expressing the recombination enzymes *Rag1* and *Rag2*, and the cyclin protein *Cdk1* during the recombination and proliferation stages of thymopoiesis. ORFTOCs were formed with TECs cultured as organoids originating from E16.5 thymi, with the EpCAM-depleted fraction of cells from E13.5 thymi and with MEFs. FTOCs were from E13.5 thymi. In the Park et al. atlas, thymi span from E14.5 to 4-6 weeks for the stromal dataset and are 4-week-old for the T-cell dataset. aDC, activated dendritic cells; commit, commitment; DC, dendritic cells; Epi, epithelial; IELpA, intestinal intraepithelial lymphocytes precursor A; IELpB, intestinal intraepithelial lymphocytes precursor B; Imm, immune; ISP, intermediate single positive; Mac, macrophages; Mono, monocytes; NKT, natural killer T; (P), proliferative; pDC, plasmacytoid dendritic cells; Prolif, proliferative; (Q), quiescent; sel, selected; TSP, thymus-seeding progenitors.

To characterize further the cell types in ORFTOCs, we profiled them and FTOC controls by single-cell RNA sequencing (scRNAseq) (Fig. 3A). This analysis revealed three main clusters corresponding to the epithelial, immune and mesenchymal compartments of ORFTOCs (Fig. 3B). Unsupervised clustering identified seven main clusters of epithelial cells (Fig. S3A), which we annotated according to *in vivo* datasets (Baran-Gale et al., 2020; Bautista et al., 2021; Bornstein et al., 2018; Gao et al., 2022; Nusser et al., 2022; Park et al., 2020): ‘early cTECs’, ‘cTECs’, ‘early mTECs’, ‘pre-Aire mTECs’, ‘Aire and Spink5 mTECs’, ‘tuft-like mTECs’ and ‘adult bipotent progenitor-like’ (Fig. S3B). For the immune cells, clusters covered the main T-cell developmental stages defined *in vivo* (Cordes et al., 2022; Luis et al., 2016; Mingueneau et al., 2013; Park et al., 2020; Rothenberg, 2021; Zhou et al., 2019), spanning from progenitors to mature T cells (Fig. S3C,D). Both ORFTOCs and FTOCs contributed to all subpopulations (Fig. 3C,D), suggesting that ORFTOCs faithfully recapitulate the different cell types present in FTOC controls. The biggest differences were observed for clusters representing cTECs and early stages of T-cell development: (1) more ‘early cTECs’ were present in FTOCs and (2) more ‘cTECs’ and ‘thymus-seeding progenitor (TSP) to DN early 1’ and ‘TSP to DN early 2’ cells were present in ORFTOCs.

To compare our *in vitro* populations with the in *vivo* thymus, we aligned our clusters to the mouse dataset of the reference atlas produced by Park et al. (2020) (Fig. 3E,F). We found strong overlap for most epithelial cell types (Fig. 3E), with the cTECs aligning together and most *in vitro* mTEC clusters matching their *in vivo* counterparts. However, the ‘adult bipotent progenitor-like’ cluster was smaller *in vivo* compared with *in vitro*. Immune clusters from our dataset also matched clusters defined for *in vivo* populations (Fig. 3F), especially from the ‘DP blast’/‘DP (P)’ stage onwards and, most importantly, for the CD4 and CD8 stages (mature T cells).

Besides gene expression, we studied *in vitro* TCR recombination dynamics by V(D)J (variable–diversity–joining) sequencing, allowing us to map productive T cells bearing both TCR chains on the immune uniform manifold approximation and projection (UMAP) (Fig. 3G). The quantification of productive chains presenting all V(D)J regions showed that the recombination of the TCRβ (TRB) and -α (TRA) chains were mostly achieved prior to and at the DP stage, respectively (Fig. 3H), similarly to the Park et al. dataset. In addition, thymocytes underwent proliferation (marked by high *Cdk1* expression) in between the recombination stages (marked by high *Rag1* and *Rag2* expression) (Fig. 3I), which also aligns with *in vivo* data (Park et al., 2020; Rothenberg, 2021).

Taken together, these results show the transcriptional similarity of ORFTOCs and FTOCs and that ORFTOCs preserve *in vivo*-like TEC diversity and T-cell development.

### ORFTOCs show a thymus-like ability to attract new T-cell progenitors and improved epithelial organization upon *in vivo* grafting

**Fig. 4.**
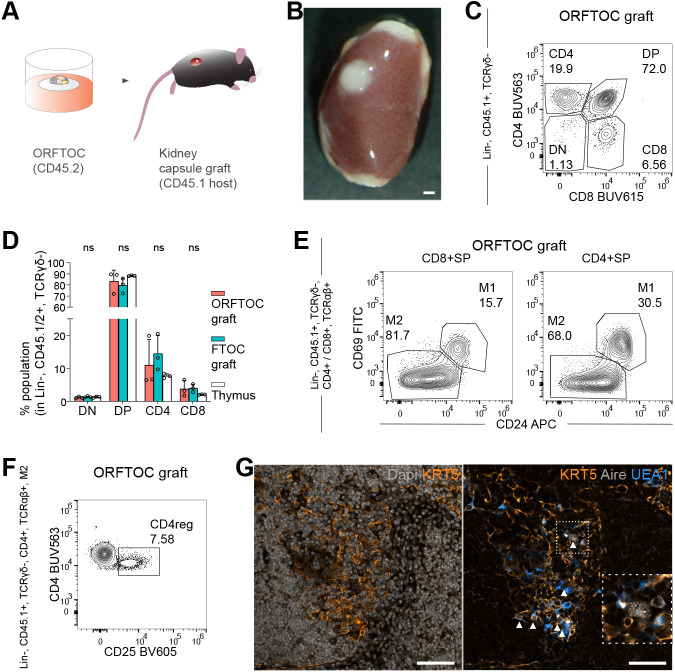
**ORFTOCs show a thymus-like ability to attract new T-cell progenitors and improved epithelial organization upon *in vivo* grafting.** (A) Experimental design for the grafting of ORFTOCs under the kidney capsule. (B) Widefield image of an ORFTOC graft after 5 weeks. (C) Flow cytometry plot showing host thymocyte development in ORFTOC grafts. Lin, Lineage. (D) Proportion of the major thymocyte subpopulations in ORFTOC grafts and controls (FTOC grafts and thymi). ns, not significant (*P*>0.05) (one-way ANOVA with Tukey's multiple comparison test for each subpopulation between conditions; *n*=3 grafts/mice for each condition). Bar graph shows mean±s.d. and individual datapoints. (E,F) Flow cytometry plots. (E) Presence of two separate post-selection stages (M1 and M2) within the CD8^+^ and CD4^+^ SP populations in ORFTOC grafts. (F) The M2 population of ORFTOC grafts contains CD4 regulatory T cells (CD4reg). Gating strategy is indicated on the left for all flow cytometry plots. (G) Immunofluorescence images of an ORFTOC graft. Left: Medullary cells (KRT5; amber) are present in the less-dense area (DAPI; grey). Right: The medullary region also contains UEA1-reactive (azure) and Aire-positive (grey, highlighted with arrowhead) cells. Inset shows the boxed area at higher magnification. ORFTOCs were formed with TECs cultured as organoids originating from E16.5 thymi, with the EpCAM-depleted fraction of cells from E13.5 thymi and with MEFs. FTOCs were from E13.5 thymi. Scale bars: 1 mm (B); 100 μm (G).

The thymus continuously attracts bone marrow-derived haematopoietic precursors and commits them to the T-cell lineage (Lai and Kondo, 2007; Lavaert et al., 2020). To test whether ORFTOCs retain this crucial capacity, we transplanted them under the kidney capsule of syngeneic CD45.1 recipient mice (Fig. 4A). After 5 weeks, all grafts developed into sizeable thymus-like tissues (4/4 ORFTOCs, 4/4 FTOC controls; see Fig. 4B for an example). By flow cytometry (Fig. S4A,B), we identified all major thymocyte populations (DN, DP, CD4, CD8) in ORFTOC grafts (Fig. 4C, Fig. S4C), and their proportions were comparable to controls (FTOC grafts and thymi) (Fig. 4D). This result demonstrated that ORFTOC grafts support normal TCRαβ T-cell lineage maturation. Further analyses (Fig. S4B,D) detected thymocytes at the DN3-to-DN4 transition at the time of ORFTOC graft retrieval, attesting to successful β-selection (Rothenberg, 2021). In addition, DP thymocytes expressing CD69, which indicates positive selection-induced TCR signalling (Steier et al., 2023), were present (Fig. S4B,E). Together, these results illustrate the ability of ORFTOC grafts to continuously attract and select blood-borne T-cell progenitors. Finally, the presence of M1 and M2 CD45.1^+^ mature thymocytes (Fig. 4E, S4B,F) and of regulatory T cells (Fig. 4F, Fig. S4B,F) showed the capacity of ORFTOC grafts to generate mature CD4^+^ and CD8^+^ T cells, impose their post-selection maturation and select T cells with a regulatory phenotype.

In histological sections, ORFTOC grafts, similar to FTOC grafts, displayed the characteristic differences in cellular densities between cortical and medullary areas seen in the native thymus (Gordon and Manley, 2011) (Fig. S4H,I). The medullary fate of the less-dense regions was confirmed by immunostaining with the lectin UEA1 and they also contained Aire-positive cells (Fig. 4G). These regions thus appeared not only larger but also more mature compared with those observed after *in vitro* culture only (Fig. 2I), likely as a result of continuous seeding with new T-cell progenitors and prolonged crosstalk with immune cells (Irla et al., 2010).

In conclusion, kidney capsule transplants showed that organoid-derived TECs in ORFTOCs have (1) the capacity to mature and reach an organization resembling the native thymus and (2) the long-term ability to attract T-cell progenitors and mediate physiological T-cell development.

## DISCUSSION

In this study, we showed that stromal cells of a lymphoid organ, namely epithelial cells of the thymus, can be cultured as 3D organoids in a similar manner to cells from other endoderm-derived organs. We established TEC-specific culture conditions, characterized the organoids, and demonstrated their superiority in maintaining TEC marker expression compared with conventional 2D cultures. Reaggregating TECs from organoid cultures with T-cell progenitors proved their functional ability to mediate thymopoiesis. TEC and T-cell populations in reaggregates resembled the native cell types, and T-cell development was recapitulated in a physiological manner. Finally, kidney capsule transplants demonstrated the long-term capability of organoid reaggregates to attract new T-cell progenitors and mediate their entire development.

Overall, applying organoid culture methods to TECs has addressed a long-standing challenge in the thymus field and established the required conditions to grow TECs independently of other cell types in a way that maintains their thymopoietic ability. FGF7, which was previously shown to act through the Ras/MAPK and PI3 K/Akt/mTOR signalling cascades (Rossi et al., 2007), was important to promote organoid development. Although thymic epithelial organoids recapitulate many key organoid features, such as cell population diversity and the possibility to be expanded and passaged, maintaining their functionality in the long term remained challenging. This is probably linked to some niche factors being missing in the current relatively minimal culture conditions, which over time either prevent the maintenance of functionality overall or result in the enrichment of specific subsets that might lack functionality (Gao et al., 2022). While this article was under revision, another study generating thymic epithelial organoids was published (Lim et al., 2024) and addressed this challenge by first expanding and then differentiating the organoids. Whereas we aimed at understanding the minimal requirements to grow thymic epithelial organoids, their media were more complex and contained additional factors that have been applied to generate other endoderm-derived epithelial organoids previously as well as RANK ligand and retinoic acid. The difference in medium composition and perhaps the fact that they started by plating minced thymic tissue without sorting also allowed them to generate organoids from adult TECs, whereas our study is limited to embryonic and early postnatal cells.

Our work, however, demonstrates that most of the cells in the embryonic thymus have the potential to form organoids. Recent scRNAseq analyses (Baran-Gale et al., 2020; Gao et al., 2022; Givony et al., 2023; Michelson et al., 2022; Nusser et al., 2022; Ragazzini et al., 2023) have revealed unforeseen heterogeneity among TECs and TEC progenitors, and progenitor–progeny relationships remain to be fully elucidated. By sorting different TEC subtypes and expanding them as organoids, our approach might help to shed light onto these relationships (at least *in vitro*). It might also allow to test the potential of specific populations to mediate T-cell development upon reaggregation with T-cell progenitors. Up to now, we only reaggregated total TECs (EpCAM positive) with T-cell progenitors, but TECs are already diverse at E16.5. Given that a sufficient number of cells for reaggregation can only be reached by pooling dissociated organoids, we cannot exclude the influence of inter-clonal heterogeneity on the functional potential of organoid-cultured TECs.

Further functional characterization could also be performed for ORFTOC-generated T cells. Indeed, we could not detect a population of CD45.2 ORFTOC-derived T cells in the periphery of the graft recipient. We believe that this is due to a dilution effect, as T cells originating from the graft are competing with those from the endogenous thymus. Further grafting experiments in nude mice would be required to study tolerance induction by ORFTOC-derived T cells as well as their ability to mount an immune response.

Merging the organoid and the thymus fields nevertheless opens up new opportunities to gain insights into TEC biology and to study T-cell development *in vitro* in a physiological manner. As TECs undergo deterioration during ageing and different medical conditions, the newly generated thymic epithelial organoids might also pave the way for future thymus regeneration strategies. Finally, thymic epithelial organoids are, to the best of our knowledge, the first bona fide organoids originating from the stromal compartment of a lymphoid organ.

## MATERIALS AND METHODS

### Mice

C57BL/6J P0 mouse bodies were obtained through the EPFL Organ/Tissue Sharing Program (OptiMice) and were a kind gift from the Lashuel laboratory (EPFL, Switzerland). For all other *in vitro* experiments, C57BL/6J mice were purchased from Charles River France and maintained in the EPFL animal facility until use. For grafting experiments, Ly5.1 and C57BL/6J mice were bred and maintained in the mouse facility of the Department of Biomedicine at the University of Basel, Switzerland. For timed mating, noon of the day of the vaginal plug was considered as E0.5. Mice were housed in individual cages at 23±1°C with a 12 h light/dark cycle, and supplied with food and water *ad libitum*. All animal work was conducted in accordance with Swiss national guidelines, reviewed and approved by the Cantonal Veterinary Offices of Vaud and of Basel-Stadt (license numbers VD3035.1, VD3823, VD3694 and BS2321).

### Isolation of TECs

E16.5 embryonic and P0 neonatal thymi were dissected and collected in Eppendorf tubes containing FACS buffer [PBS (Gibco,10010-015)+2% fetal bovine serum (FBS; Thermo Fisher Scientific, 26140079)]. Lobes were rinsed with PBS and digested with 475 µl TrypLE (Gibco, 12605-028) for 5 min at 37°C under agitation (Eppendorf, ThermoMixer C). Lobes were pipetted to promote dissociation, 25 μl DNase (Sigma-Aldrich, 10104159001; from 1 mg/ml stock) was added and the tubes were incubated for another 5 min. Lobes were again pipetted to help dissociation, TrypLE was quenched with 1 ml Adv. DMEM/F12 (Thermo Fisher Scientific, 12634028) containing 10% FBS and the cell suspension was filtered through a 40 μm strainer. The cells were pelleted and resuspended in FACS buffer. Cell suspensions from P0 thymi were processed through a CD45-depletion step using immunomagnetic beads (Miltenyi Biotec, 130-052-301; 1/10). After a 20 min incubation at 4°C, the unbound complexes were washed and the cells processed through magnetic columns (Miltenyi Biotec, 130-042-401) following the manufacturer's instruction. The CD45-depleted fraction was collected and stained for sorting. Cell suspensions were then stained for 20 min at 4°C protected from light with the antibodies listed hereafter. For total TECs: Ter119-FITC (BioLegend, 116205; 1/100), EpCAM-PE (BioLegend, 1198206; 1/80), PDGFR-α-APC (BioLegend, 135907; 1/40), PDGFR-β-ACP (BioLegend, 136007; 1/40), CD31-PE-Cy7 (BioLegend, 102524; 1/160), MHCII-APC/Fire750 (BioLegend, 107651; 1/160), CD45-Pacific Blue (BioLegend, 103126; 1/200).

For cTECs and mTECs, cells were first incubated with UEA1 (Vector Laboratories B-1065; 1/800), then washed and incubated with CD45-FITC (BioLegend, 103108; 1/100), Ter119-FITC (BioLegend, 116205; 1/100) (together referred as Lineage), EpCAM-PE (BioLegend, 1198206; 1/80), Streptavidin-BV711 (BioLegend, 405241; 1/80) and CD205-APC (Thermo Fisher Scientific, 17-2051-82; 1/400).

DAPI (Tocris, 4748; 0.5 μg/ml) was used to exclude dead cells. After staining, the antibodies were washed and the cells resuspended in FACS buffer for sorting using an Aria Fusion (BD Biosciences). The sorting strategy for isolating EpCAM^+^ TECs is shown in Fig. S1A. For cTECs and mTECs, live cells were gated on EpCAM^+^, Lineage^−^ and separated into cTECs and mTECs based on CD205 and UEA1 staining (Fig. S1E). Sorted cells were collected in TEC medium (see below) containing 2% FBS and 2.5 μM thiazovivin (Stemgen, AMS.04-0017).

### CellTrace labelling

Sorted TECs were equally divided into two sets and labelled with either CellTrace Oregon Green 488 (carboxy-DFFDA SE) (Thermo Fisher Scientific, C34555) or CellTrace Far Red (Thermo Fisher Scientific, C34572) according to the manufacturer's instructions. After washes, labelled cells were mixed in a 1:1 ratio and plated as described below.

### Thymic epithelial organoid culture

Sorted TECs were embedded in growth factor-reduced Matrigel (Corning, 356231) (∼1.55×10^4^ cells per 20 ml drop) and plated in 24-well plates (Corning, 353047; or Ibidi, 82426). After Matrigel polymerization, TEC medium was added. TEC medium consisted of organoid basal medium [Advanced DMEM/F-12 supplemented with 1× GlutaMAX (Thermo Fisher Scientific, 35050038), 10 mM HEPES (Thermo Fisher Scientific, 15630056), 100 μg ml^−1^ Penicillin–Streptomycin (Thermo Fisher Scientific, 15140122), 1× B-27 supplement (Thermo Fisher Scientific, 17504001), 1× N2 supplement (Thermo Fisher Scientific, 17502001), 1 mM N-acetylcysteine (Sigma-Aldrich, A9165)] plus 100 ng ml^−1^ FGF7 (Peprotech, 100-19). For the first 2 days, 2.5 μM thiazovivin was also added to the medium. Medium was changed every second day. Organoids were cultured at 37°C with 5% CO_2_.

### Organoid proliferation assays and organoid-forming efficiency

Sorted TECs were embedded in 10 μl Matrigel drops (∼7.5×10^3^ cells/drop) in a 48-well plate (Corning, 353078). On the day of seeding (day 0), at days 1, 3, 5 and 7, 220 μM resazurin (Sigma-Aldrich, R7017) was added to organoid basal medium and incubated with the cells for 4 h at 37°C. Afterwards, the resazurin-containing medium was collected and replaced by fresh TEC medium with or without FGF7. Organoid proliferation was estimated by measuring the reduction of resazurin to fluorescent resorufin in the medium using a Tecan Infinite F500 microplate reader with 560 nm excitation and 590 nm emission filters. For analysis, data were normalized from minimum to maximum.

Organoid-forming efficiency was estimated by calculating the ratio of the number of cells at day 1 to the number of organoids at day 7 in the same field of view.

### Bulk transcriptome profiling

Sorted TECs were culture as indicated above. As controls, sorted TECs from E16.5 embryos were either directly lysed in RLT buffer (QIAGEN, 74004) containing 40 mM DTT (ITW Reagents, A2948) or cultured in 2D on plates coated with 6 μg/ml laminin (R&D Systems, 3446-005-01). Cultures were maintained in TEC medium. Organoids were collected in cold PBS to dissolve Matrigel and then lysed in RLT buffer with DTT. They were collected after 3 and 7 days. Cells cultured in 2D were directly collected in RLT buffer with DTT. They were collected once a confluent monolayer formed, after 3 days, as prolonged culture in these conditions lead to cell detachment and death. RNA was extracted using the QIAGEN RNeasy Micro Kit (QIAGEN, 74004) according to the manufacturer's instructions. Purified RNA was quality checked using a TapeStation 4200 (Agilent), and 88 ng were used for QuantSeq 3′ mRNA-Seq library construction according to the manufacturer's instructions (Lexogen, 015.96). Libraries were quality checked using a Fragment Analyzer (Agilent) and were sequenced in a NextSeq 500 (Illumina) using NextSeq v2.5 chemistry with Illumina protocol #15048776. Reads were aligned to the mouse genome (GRCm39) using star (version 2.7.0e). R (version 4.1.2) was used to perform differential expression analyses. Count values were imported and processed using edgeR (Robinson et al., 2010). Expression values were normalized using the trimmed mean of M values (TMM) method and genes expressed at low levels (<1 count per million) and genes present in fewer than three samples were filtered out. Differentially expressed genes were identified using linear models (Limma-Voom) (Smyth et al., 2018), and *P*-values were adjusted for multiple comparisons by applying the Benjamini–Hochberg correction method (Reiner et al., 2003). Voom expression values were used for hierarchical clustering using the function hclust (https://www.rdocumentation.org/packages/stats/versions/3.6.2/topics/hclust) with default parameters, and for heatmap generation. *Foxn1* target genes corresponded to the Top 30 list from Žuklys et al. (2016). Some genes were filtered out owing to the aforementioned exclusion criteria. Single sample gene set enrichment analysis (GSEA) (Subramanian et al., 2005) was used to score the E2F targets hallmark proliferation gene set (Howe et al., 2018; Liberzon et al., 2015) between samples.

### Whole-mount immunofluorescence staining

Organoid samples were fixed in 4% paraformaldehyde (Thermo Fisher Scientific, 15434389) in PBS for 30 min at room temperature and subsequently washed with PBS. Samples were permeabilized in 0.2% Triton X-100 (Sigma-Aldrich, T8787), 0.3 M glycine (Invitrogen, 15527-013) in PBS for 30 min at room temperature and blocked in 10% serum [goat (Thermo Fisher Scientific, 16210064) or donkey (Abcam, ab7475)], 0.01% Triton X-100 and 0.3 M glycine in PBS for 4 h at room temperature. Samples were then incubated with primary antibodies overnight at 4°C, washed with PBS, incubated with secondary antibodies overnight at 4°C, and washed with PBS. Mounting was carried out with Fluoromount-G (SouthernBiotech, 0100-01). The following primary and secondary antibodies were used: MHCII-Biotin (BioLegend, 107603; 1/200), UEA1 (Vector Laboratories, B-1065; 1/500), keratin 5 (BioLegend, 905501; 1/200), keratin 8 (Abcam, ab53280; 1/200), Ki67 (BD Pharmingen, 550609; 1/200), EpCAM-APC (Invitrogen, 17-5791-82; 1/200), Streptavidin Alexa 488 (Thermo Fisher Scientific, S-11223; 1/500), Streptavidin Alexa 647 (Thermo Fisher Scientific, S-21374; 1/500), goat anti-rat Alexa 647 (Thermo Fisher Scientific, A-21247; 1/500), donkey anti-mouse Alexa 568 (Thermo Fisher Scientific, A-10037; 1/500), donkey anti-rabbit Alexa 488 (Thermo Fisher Scientific, A-21206; 1/500), donkey anti-rabbit Alexa 568 (Thermo Fisher Scientific, A-11077; 1/500). DAPI (Tocris, 4748, 1 mg/ml) was used to stain nuclei.

### Reaggregate culture

E13.5 embryonic thymi were dissected and collected in Eppendorf tubes containing FACS buffer. Lobes were rinsed with PBS and digested with 475 μl TrypLE and 25 μl DNase (from 1 mg/ml stock) for 5 min under agitation. Lobes were pipetted to help dissociation and TrypLE was quenched with 1 ml Adv. DMEM/F12 containing 10% FBS. The cells were pelleted and resuspended in FACS buffer for immunomagnetic cell separation with EpCAM-conjugated beads (Miltenyi Biotec, 130-105-958; 1/4). After a 20 min incubation at 4°C, the unbound complexes were washed and the cells processed through magnetic columns (Miltenyi Biotec, 130-042-401) following the manufacturer's instruction. The EpCAM-depleted fraction was collected and used to prepare reaggregates with dissociated thymic epithelial organoids and MEFs.

Thymic epithelial organoids at day 7 of culture were collected in cold Advanced DMEM/F-12 supplemented with 1× GlutaMAX, 10 mM HEPES and 100 μg ml^−1^ Penicillin–Streptomycin. Organoids were pelleted and digested with 950 μl TrypLE and 50 μl DNase (from 1 mg/ml) for 5 min at 37°C. Organoids were pipetted to improve dissociation. In case digestion was insufficient, organoids were further digested for 5 min with Trypsin+0.25% EDTA (Gibco, 25200-072) at 37°C and pipetted until the obtention of a single-cell suspension. Dissociation was quenched with Adv. DMEM/F12 containing 10% FBS and the cells pelleted.

Wild-type MEFs were a kind gift from the Blackburn laboratory (Centre for Regenerative Medicine, Institute for Regeneration and Repair, School of Biological Sciences, University of Edinburgh, Edinburgh, UK). MEFs were cultured in Advanced DMEM/F-12 supplemented with 1× GlutaMAX, 1× Non-Essential Amino Acids (Gibco, 11140035), 100 μg ml^−1^ Penicillin–Streptomycin and 10% FBS on gelatin-coated dishes (0.1% gelatin in H_2_O) (Sigma-Aldrich, G1890). MEFs were harvested using Trypsin EDTA 0.25% for 2 min at 37°C. Dissociation was quenched with Adv. DMEM/F12 containing 10% FBS and the cells pelleted.

For reaggregates using adult DN1 thymocytes as input population, adult thymi were dissected from 4-week-old female C57BL/6J mice. Thymi were cut into small pieces with a scalpel to liberate thymocytes, which were filtered to a single-cell suspension with a 40 μm strainer. Cells were incubated with APC anti-mouse CD8a Antibody (BioLegend, 100711; 1/50) for 20 min at 4°C in FACS buffer and washed. Cells were then incubated with anti-APC magnetic beads (Miltenyi Biotec, 130-090-855; 1/4) for 20 min at 4°C. The unbound beads were washed away and the cells processed through magnetic columns following the manufacturer's instructions. The APC-depleted fraction was collected and used for staining with the following antibodies: Ter119-FITC (BioLegend, 116205; 1/800), Cd45R-FITC (BioLegend, 103205; 1/800), CD11b-FITC (Thermo Fisher Scientific, 11-0112-82; 1/800), Ly-6G-FITC (BioLegend, 108405; 1/800), Cd11C-FITC (BioLegend, 117306; 1/800), NK-1.1-FITC (BioLegend, 108705; 1/800), CD3-FITC (BioLegend, 100306), CD4-FITC (BioLegend, 100510), (together referred to as Lineage) CD45-Pacific Blue (BioLegend, 103126; 1/200) or CD45-AF700 (BioLegend, 103127; 1/160), CD44-PE (BioLegend, 103008; 1/160), CD25-BV711 (BioLegend, 102049; 1/160) and DAPI (Tocris, 4748; 0.5 μg/ml). After staining, the antibodies were washed and the cells resuspended in FACS buffer for sorting using an Aria Fusion (BD Biosciences). The sorting strategy for isolating DN1 thymocytes was gating on cells, single cells, live cells, CD45^+^ Lineage^−^ cells, and CD44^+^ CD25^−^ cells. DN1 thymocytes were collected in ORFTOC medium (see below).

ORFTOCs were prepared as previously described (Sheridan et al., 2009). Briefly, the cell suspension for each ORFTOC typically contained 10^5^ EpCAM-depleted cells, 10^5^ thymic epithelial organoid cells and 10^5^ MEFs (or 10^5^ thymic epithelial organoid cells, 4×10^4^ DN1 thymocytes and 10^5^ MEFs). These cells were transferred to an Eppendorf tube and pelleted. The pellet was resuspended in 60 μl of the medium used for culture, and transferred to a tip sealed with Parafilm inside a 15 ml Falcon tube. Cells were pelleted inside the tip for 5 min at 470 ***g***. The pellet was then gently extruded on top of a filter membrane (Merck, ATTP01300) floating on culture medium in 24-well plate. ORFTOC culture medium consisted of advanced DMEM/F-12 supplemented with 1× GlutaMAX, 1× Non-Essential Amino Acids, 100 μg ml^−1^ Penicillin–Streptomycin, 2% FBS and 100 ng/ml FGF7. 2.5 μM Thiazovivin was added for the first 2 days of culture and half of the medium volume was changed every second day.

Controls in which one of the cell population was absent were made the same way. For FTOC controls, E13.5 dissected lobes were directly placed on top of a filter membrane and also cultured in ORFTOC medium.

All cultures were maintained at 37°C with 5% CO_2_.

### Flow cytometry analysis of ORFTOCs and FTOCs

After 6, 13 or 20 days in culture, ORFTOCs, FTOCs, reaggregates with DN1 thymocytes and control reaggregates were gently detached from the filter membrane by pipetting and transferred to Eppendorf tubes, together with the culture medium to collect recently emigrated T cells. Samples were pelleted, rinsed with PBS and digested with 200 μl TrypLE for 10 min at 37° C with agitation on an Eppendorf shaker (800 rpm). Dissociation was quenched with 1 ml Adv. DMEM/F12 containing 10% FBS and the cells pelleted. Cells were resuspended in FACS buffer for staining. The cells were incubated for 20 min with the following antibodies: Ter119-FITC (BioLegend, 116205; 1/800), Cd45R-FITC (BioLegend, 103205; 1/800), CD11b-FITC (Thermo Fisher Scientific, 11-0112-82; 1/800), Ly-6G-FITC (BioLegend, 108405; 1/800), Cd11C-FITC (BioLegend, 117306; 1/800), NK-1.1-FITC (BioLegend, 108705; 1/800) (together referred to as Lineage), CD44-PE (BioLegend, 103008; 1/160), CD69-APC (BioLegend, 104513; 1/160), CD4-BV605 (BioLegend, 100548; 1/40), CD3-PerCP/Cy5.5 (BioLegend, 100327; 1/160), CD8-PE/Cy7 (BioLegend, 100722; 1/160), CD25-BV711 (BioLegend, 102049; 1/160), CD45-AF700 (BioLegend, 103127; 1/160), TCRβ-BV421 (BioLegend, 109230; 1/80) and DAPI (Tocris, 4748, 0.5 μg/ml). After staining, the antibodies were washed and the cells resuspended in FACS buffer for analysing using a LSR Fortessa Cytometer (BD Biosciences). The gating strategy for analysis is shown in Fig. S2B. Beads (UltraComp; Thermo Fisher Scientific, 01-3333-42) were used for single-colour staining for compensation. Gates were based on T cells extracted from young adult mice. Flow cytometry data were analysed using FlowJo (BD Biosciences, version 10.9.0).

### Single-cell transcriptome profiling

After 13 days in culture, ORFTOC and FTOC samples were collected and dissociated as described for flow cytometry analysis. After dissociation, two ORFTOC samples and two FTOC samples were pooled, respectively. For each pool, 500,000 cells were incubated with 1 μl TotalSeq Antibody (HTO) (BioLegend, 155863 and 155861) in 50 μl FACS buffer for 30 min on ice. Antibodies were washed twice with FACS buffer and the single-cell suspensions filtered through a 40 μm strainer. After cell count, samples were mixed in a 1:1 ratio and processed using Chromium Next GEM Single Cell 5′ Reagent Kits v2 (Dual Index) with Feature Barcode technology for Cell Surface Protein & Immune Receptor Mapping reagents (10x Genomics, PN-1000265, PN-1000256, PN-1000190, PN-1000287, PN-1000215 and PN-100025) following the manufacturer's instructions. The Single Cell Mouse TCR amplification Kit (10x Genomics, 1000254) was used to prepare TCR libraries. Sequencing was carried out using NovaSeq v1.5 STD (Illumina protocol #1000000106351 v03) for around 100,000 reads per cell. The reads were aligned using Cell Ranger v6.1.2 to the mouse genome (mm10). Raw count matrices were imported into R and analysed using Seurat v4.2.0 (Hao et al., 2021). HTO with fewer than 100 features and fewer than 1 count were discarded. Cells with fewer than 600 features, <0.4 or >10% mitochondrial genes were discarded. Demultiplexing was performed using HTODemux with standard parameters. Doublets were removed using recoverDoublets from scDblFinder package (Germain et al., 2022) and based on doublets identified from HTOs. Data were normalized using SCTransform and with cell cycle score as variable to regress. The three clusters representing the main cell types were obtained using principal component analysis and UMAP with 18 dimensions and a resolution of 0.005. Each cell type was then subset and thresholded based on *EpCAM*, *Ptprc* and *Pdgfra* expression. Epithelial clusters were identified using 18 dimensions and a resolution of 0.4, leading to seven clusters that were named based on markers from previous datasets (Baran-Gale et al., 2020; Bautista et al., 2021; Bornstein et al., 2018; Gao et al., 2022; Kernfeld et al., 2018; Park et al., 2020). Immune clusters were identified using 18 dimensions and a resolution of 3. Immune clusters were further merged to obtain 14 clusters representing main T-cell developmental stages based on markers from previous datasets (Cordes et al., 2022; Mingueneau et al., 2013; Park et al., 2020; Rothenberg, 2021). The number of cells per cluster in both FTOC and RFTOC samples were calculated to show HTO repartition between both samples. TCR analysis was conducted using scRepertoire (Borcherding et al., 2020). Filtered contig output from Cell Ranger was used as input and added to immune cell metadata. Productive cells with both TRA and TRB chains were plotted on the UMAP, and the percentage of productive cells (either at least TRB chain with no NA and no double chain, or both TRA and TRB chains with no NA and double chain accepted only for TRA) per cluster was calculated. The mouse samples from the dataset from Park et al. (2020) were used for alignment. H5ad files were converted to Seurat objects. TECs (E14.5, E18.5, 6 days and 4 weeks old) were subset from the stromal dataset used in the study (GSE103967). Four-week-old T cells from the Park et al. dataset were subset from the mouse total dataset (doi:10.5281/zenodo.3572422). Alignment was performed using SCTransform and canonical correlation analysis (CCA) with the Park dataset labelled as reference and otherwise default parameters.

### Kidney capsule grafting and analysis

Transplants were perfomed after 5 days of culture. ORFTOCs were grafted in CD45.1 host mice and FTOC controls in CD45.2 host mice. Mice were treated with the analgesic carprofen (10 mg/kg in drinking water) 12-24 h prior to transplantation. Mice were anaesthetized with Ketalar/Rompun (100 mg/kg ketamine and 20 mg/kg xylazine, intraperitoneal). Lacrinorm eye gel (Bausch+Lomb) was administered to avoid dehydration of the cornea during the procedure. Anaesthetized mice were shaved laterally and disinfected using Betadine. The surgery was performed on a heating pad in order to minimize body temperature drop. A small incision of approximately 1 cm was made first on the skin and then in the peritoneum. By pulling at the posterior fat of the kidney with forceps, the kidney was exposed outside of the peritoneum and kept wet with PBS. Under the microscope, an incision and a channel were made with watchmaker forceps on the kidney capsule's membrane and one ORFTOC or FTOC was placed under the membrane. After positioning the kidney back into the peritoneum, the wound was closed with two stitches (resorbable suture material 5/0; Polyactin 910; RB-1 plus; Johnson & Johnson). The skin opening was closed with staples, which were removed 7-10 days later. An analgesic (Temgesic, buprenorphine 0.1 mg/kg, subcutaneous) was administered at the end of the procedure followed by continuous treatment of transplanted mice by carprofen (10 mg/kg in drinking water) for 3 days. After the transplants, mice were monitored daily and weighed every second day to confirm their wellbeing. Grafts were analysed 5 weeks after transplantation.

At the time of analysis, mice were euthanized with CO_2_ and kidneys retrieved. Grafts were separated from the kidney under the microscope. Native thymi were used as controls. To collect T cells, grafts and thymi were mechanically dissociated by pipetting in FACS buffer. Single-cell suspensions were then stained with Zombie NIR (BioLegend, 423105; 1/1000) for 30 min at 4°C. Samples were washed with FACS buffer and incubated with the following Lineage antibodies for 30 min at 4°C: CD11b Biotin (BioLegend, 101204; 1/1000), CD11c Biotin (BioLegend, 117304; 1/1000), CD19 Biotin (BioLegend, 101504; 1/1000), DX5 Biotin (BioLegend, 108904; 1/1000), MHCII Biotin (BioLegend, 116404; 1/1000), GR1 Biotin (BioLegend, 108404; 1/1000), F4/80 Biotin (BioLegend, 123100; 1/1000), Ter119 Biotin (BioLegend, 116204; 1/1000) and NK-1.1 Biotin (BioLegend, 108704; 1/1000). After washes, samples were incubated with the following antibodies for 30 min at 4°C: CD45.1-PerCP-Cy5.5 (BioLegend, 110728; 1/500), CD45.2-BV650 (BioLegend, 109836; 1/200), CD4-BUV563 (Thermo Fisher Scientific, 365-0042-82; 1/1000), CD8-BUV615 (Thermo Fisher Scientific, 366-0081-82; 1/500), TCRαβ-PE-Dazzle594 (BioLegend, 109220; 1/500), TCRγδ-PE (BioLegend, 118108; 1/500), CD69-FITC (BioLegend, 104506; 1/500), CD24-APC (BioLegend, C101814; 1/1000), CD44-BV785 (BioLegend, 103059; 1/500), Ckit-BUV737 (Thermo Fisher Scientific, 367-1171-82; 1/200), CD71-PE-Cy7 (BioLegend, 113812; 1/200), Sca1-BUV395 (Thermo Fisher Scientific, 363-5981-82; 1/500), CD25-BV605 (BioLegend, 102036; 1/500), CD5-APC-eF780 (Thermo Fisher Scientific, 47-0015-82; 1/500) and Streptavidin-BV510 (Biolegend, 405234; 1/500). After final washes, samples were resuspended in FACS buffer and analysed on an Aurora flow cytometer (Cytek Biosciences). Flow cytometry data were then analysed using FlowJo (version 10.9.0).

### Sectioning, immunofluorescence staining and RNAscope on sections

Organoids, ORFTOCs, FTOCs and grafts were fixed in 4% paraformaldehyde in PBS for 30 min at room temperature (organoids) to overnight at 4°C (ORFTOCs, FTOCs, grafts). Samples were then washed with PBS and either processed for cryosectioning or for paraffin embedding.

For cryosectioning, samples were incubated in 30% (W/V) sucrose (Sigma-Aldrich, S1888) in PBS until the sample sank. Subsequently, samples were incubated for 12 h in a mixture of Cryomatrix (Epredia, 6769006) and 30% sucrose (Sigma-Aldrich, 84097) (mixing ratio 50/50), followed by a 12 h incubation in pure Cryomatrix. The samples were then embedded in a tissue mould, frozen on dry ice or in isopentane cooled by surrounding liquid nitrogen, and 10-µm-thick sections were cut at −20°C using a CM3050S cryostat (Leica Biosystems).

For paraffin embedding, organoid, ORFTOC and FTOC samples were embedded in HistoGel (Thermo Fisher Scientific, HG-4000-012) before being placed into histology cassettes. Cassettes were then processed with a Tissue-Tek VIP 6 AI Vacuum Infiltration Processor (Sakura) and embedded in paraffin, then 4 µm paraffin sections were obtained with a Leica RM2265 microtome. Slides were processed through de-waxing and antigen retrieval in citrate buffer at pH 6.0 using a heat-induced epitope retrieval PT module (Thermo Fisher Scientific) before proceeding with immunostaining.

Sections were blocked and permeabilized for 30 min in 1% bovine serum albumin (Thermo Fisher Scientific, 15260-037), 0.2% Triton X-100 in PBS and blocked for 30 min in 10% goat or donkey serum in PBS at room temperature. Primary antibodies were incubated overnight at 4°C in 1.5% donkey or goat serum in PBS. On the following day, slices were washed twice in 1% bovine serum albumin, 0.2% Triton X-100 in PBS and incubated with secondary antibodies at room temperature for 45 min. Finally, slices were washed twice in 0.2% Triton X-100 in PBS and mounted with Fluoromount-G. The following primary and secondary antibodies were used: UEA1 (Vector Laboratories, B-1065; 1/500), keratin 5 (BioLegend, 905501; 1/200), keratin 8 (Abcam, ab53280; 1/200), CD3ε (Thermo Fisher Scientific, MA5-14524; 1/200), MHCII-Biotin (BioLegend, 107603; 1/200) EpCAM-PE (BioLegend, 118206; 1/200), Aire (Thermo Fisher Scientific, 14-5934-82; 1/50), Streptavidin Alexa 488 (Thermo Fisher Scientific, S-11223; 1/500), goat anti-rat Alexa 568 (Thermo Fisher Scientific, A-11077; 1/500) and donkey anti-rabbit Alexa 647 (Thermo Fisher Scientific, A-31573; 1/500). Nuclei were stained with DAPI (Tocris, 4748, 1 μg/ml).

RNAscope Multiplex Fluorescent V2 assay (Bio-Techne, 323110) was performed according to the manufacturer's protocol. Paraffin sections were hybridized with the probes Mm-Foxn1 (Bio-Techne, 482021). Mm-3Plex probes (Bio-Techne, 320881) and 3Plex Dapb probes (Bio-Techne, 320871) were used as positive and negative controls, respectively. Probes were incubated at 40°C for 2 h, and the different channels were revealed with TSA Opal570 (Akoya Biosciences, FP1488001KT). Tissues were counterstained with DAPI and mounted with ProLong Gold Antifade Mountant (Thermo Fisher Scientific, P36930). Haematoxylin and Eosin staining was performed using a Ventana Discovery Ultra automated slide preparation system (Roche).

### Microscopy and image analysis

Live brightfield imaging was performed using a Nikon Eclipse Ti2 inverted microscope with 4×/0.13 NA, 10×/0.30 NA and 40×/0.3 NA air objectives and a DS-Qi2 camera (Nikon Corporation). Time-lapse movies were obtained with a Nikon Eclipse Ti inverted microscope system equipped with a 20×/0.45 NA air objective and a DS-Qi2 camera (Nikon Corporation). Both microscopes were controlled using the NIS-Elements AR software (Nikon Corporation). Extended depth of field (EDF) of brightfield images was calculated using a built-in NIS-Elements function. Fluorescent confocal imaging of fixed whole-mount and sections was carried out on a Leica SP8 microscope system, equipped with 20×/0.75 NA air and 40×/1.25 glycerol objectives, 405 nm, 488 nm, 552 nm and 638 nm solid state lasers, DAPI, FITC, RHOD and Y5 filter cubes, a DFC 7000 GT (Black/White) camera and a CCD greyscale chip. Sections were also imaged on a Leica DM5500 upright microscope equipped with 20×/0.7 NA air and 40×/1 NA oil objectives, a DFC 3000 (black/white) or a DMC 2900 (colour) cameras and a CCD greyscale or a CMOS colour chips, respectively. Both Leica microscopes were controlled by the Leica LAS-X software (Leica microsystems). For image processing, only standard contrast- and intensity-level adjustments were performed, using Fiji/ImageJ (NIH) (version 2.1.0/1.53c).

### Statistics

The number of replicates (*n*), the number of independent experiments or animals, the type of statistical tests performed, and the statistical significance are indicated for each graph in the figure legend. Statistical significance was analysed using either unpaired *t*-test, one-way ANOVA or its nonparametric equivalent (Kruskal–Wallis test), or two-way ANOVA. For multiple comparisons, one-way ANOVA were followed by Tukey's test, Kruskal–Wallis test by Dunn's test, and two-way ANOVA by Tukey's or Šidák's test. For unpaired *t*-test, data normality and equality of variances were previously tested with Shapiro–Wilk and F test, respectively. For one-way ANOVA, these assumptions were tested with Shapiro–Wilk and Brown–Forsythe test. Grubbs test was used to determine the presence of outliers across scRNAseq subpopulations. In all cases, values were considered significant when *P*≤0.05. Graphs show individual datapoints with mean±standard deviation (s.d.). Tests were performed using Prism (GraphPad, version 9.4.0), except Grubbs test, which was performed using the GraphPad website (https://www.graphpad.com/quickcalcs/grubbs1/). Graphs were generated using Prism.

## Supplementary Material



10.1242/develop.202853_sup1 Supplementary information

Table S1,2. Differentially expressed genes and Voom expression from bulk RNA sequencing analysis. Tables with differential expression and Voom expression for the three conditions (*In vivo*, Organoids and 2D) analysed in the article.

## References

[DEV202853C1] Alawam, A. S., Anderson, G. and Lucas, B. (2020). Generation and regeneration of thymic epithelial cells. *Front. Immunol.* 11, 858. 10.3389/fimmu.2020.00858 32457758 PMC7221188

[DEV202853C2] Anderson, G. and Jenkinson, E. J. (1998). Use of explant technology in the study of in vitro immune responses. *J. Immunol. Methods* 216, 155-163. 10.1016/S0022-1759(98)00076-3 9760221

[DEV202853C3] Anderson, G., Jenkinson, E. J., Moore, N. C. and Owen, J. J. T. (1993). MHC class II-positive epithelium and mesenchyme cells are both required for T-cell development in the thymus. *Nature* 362, 70-73. 10.1038/362070a0 8446171

[DEV202853C4] Anderson, K. L., Moore, N. C., McLoughlin, D. E. J., Jenkinson, E. J. and Owen, J. J. T. (1998). Studies on thymic epithelial cells in vitro. *Dev. Comp. Immunol.* 22, 367-377. 10.1016/S0145-305X(98)00011-1 9700465

[DEV202853C5] Asnaghi, M. A., Barthlott, T., Gullotta, F., Strusi, V., Amovilli, A., Hafen, K., Srivastava, G., Oertle, P., Toni, R., Wendt, D. et al. (2021). Thymus extracellular matrix–derived scaffolds support graft–resident thymopoiesis and long–term in vitro culture of adult thymic epithelial cells. *Adv. Funct. Mater.* 31, 2010747. 10.1002/adfm.202010747 34539304 PMC8436951

[DEV202853C6] Baran-Gale, J., Morgan, M. D., Maio, S., Dhalla, F., Calvo-Asensio, I., Deadman, M. E., Handel, A. E., Maynard, A., Chen, S., Green, F. et al. (2020). Ageing compromises mouse thymus function and remodels epithelial cell differentiation. *eLife* 9, e56221. 10.7554/eLife.56221 32840480 PMC7490013

[DEV202853C7] Bautista, J. L., Cramer, N. T., Miller, C. N., Chavez, J., Berrios, D. I., Byrnes, L. E., Germino, J., Ntranos, V., Sneddon, J. B., Burt, T. D. et al. (2021). Single-cell transcriptional profiling of human thymic stroma uncovers novel cellular heterogeneity in the thymic medulla. *Nat. Commun.* 12, 1096. 10.1038/s41467-021-21346-6 33597545 PMC7889611

[DEV202853C8] Boehm, T. and Swann, J. B. (2013). Thymus involution and regeneration: two sides of the same coin? *Nat. Rev. Immunol.* 13, 831-838. 10.1038/nri3534 24052146

[DEV202853C9] Bonfanti, P., Claudinot, S., Amici, A. W., Farley, A., Blackburn, C. C. and Barrandon, Y. (2010). Microenvironmental reprogramming of thymic epithelial cells to skin multipotent stem cells. *Nature* 466, 978-982. 10.1038/nature09269 20725041

[DEV202853C10] Borcherding, N., Bormann, N. L. and Kraus, G. (2020). scRepertoire: an R-based toolkit for single-cell immune receptor analysis. *F1000Res.* 9, 47 10.12688/f1000research.22139.1 32789006 PMC7400693

[DEV202853C11] Bornstein, C., Nevo, S., Giladi, A., Kadouri, N., Pouzolles, M., Gerbe, F., David, E., Machado, A., Chuprin, A., Tóth, B. et al. (2018). Single-cell mapping of the thymic stroma identifies IL-25-producing tuft epithelial cells. *Nature* 559, 622-626. 10.1038/s41586-018-0346-1 30022162

[DEV202853C12] Bourgine, P. E., Klein, T., Paczulla, A. M., Shimizu, T., Kunz, L., Kokkaliaris, K. D., Coutu, D. L., Lengerke, C., Skoda, R., Schroeder, T. et al. (2018). In vitro biomimetic engineering of a human hematopoietic niche with functional properties. *Proc. Natl. Acad. Sci. USA* 115, E5688-E5695. 10.1073/pnas.1805440115 29866839 PMC6016789

[DEV202853C13] Bredenkamp, N., Ulyanchenko, S., O'Neill, K. E., Manley, N. R., Vaidya, H. J. and Blackburn, C. C. (2014). An organized and functional thymus generated from FOXN1-reprogrammed fibroblasts. *Nat. Cell Biol.* 16, 902-908. 10.1038/ncb3023 25150981 PMC4153409

[DEV202853C14] Campinoti, S., Gjinovci, A., Ragazzini, R., Zanieri, L., Ariza-McNaughton, L., Catucci, M., Boeing, S., Park, J. E., Hutchinson, J. C., Muñoz-Ruiz, M. et al. (2020). Reconstitution of a functional human thymus by postnatal stromal progenitor cells and natural whole-organ scaffolds. *Nat. Commun.* 11, 6372. 10.1038/s41467-020-20082-7 33311516 PMC7732825

[DEV202853C15] Chaudhry, M. S., Velardi, E., Dudakov, J. A. and van den Brink, M. R. M. (2016). Thymus: the next (re)generation. *Immunol. Rev.* 271, 56-71. 10.1111/imr.12418 27088907 PMC4837659

[DEV202853C16] Cordes, M., Canté-Barrett, K., van den Akker, E. B., Moretti, F. A., Kiełbasa, S. M., Vloemans, S. A., Garcia-Perez, L., Teodosio, C., van Dongen, J. J. M., Pike-Overzet, K. et al. (2022). Single-cell immune profiling reveals thymus-seeding populations, T cell commitment, and multilineage development in the human thymus. *Sci. Immunol.* 7, eade0182. 10.1126/sciimmunol.ade0182 36367948

[DEV202853C17] Fan, Y., Tajima, A., Goh, S. K., Geng, X., Gualtierotti, G., Grupillo, M., Coppola, A., Bertera, S., Rudert, W. A., Banerjee, I. et al. (2015). Bioengineering thymus organoids to restore thymic function and induce donor-specific immune tolerance to allografts. *Mol. Ther.* 23, 1262-1277. 10.1038/mt.2015.77 25903472 PMC4817796

[DEV202853C18] Gao, H., Cao, M., Deng, K., Yang, Y., Song, J., Ni, M., Xie, C., Fan, W., Ou, C., Huang, D. et al. (2022). The lineage differentiation and dynamic heterogeneity of thymic epithelial cells during thymus organogenesis. *Front. Immunol.* 13, 805451. 10.3389/fimmu.2022.805451 35273595 PMC8901506

[DEV202853C19] Germain, P. L., Robinson, M. D., Lun, A., Garcia Meixide, C. and Macnair, W. (2022). Doublet identification in single-cell sequencing data using scDblFinder. *F1000Res.* 10, 979. 10.12688/f1000research.73600.2 PMC9204188 35814628

[DEV202853C20] Giger, S., Hofer, M., Miljkovic-Licina, M., Hoehnel, S., Brandenberg, N., Guiet, R., Ehrbar, M., Kleiner, E., Gegenschatz-Schmid, K., Matthes, T. et al. (2022). Microarrayed human bone marrow organoids for modeling blood stem cell dynamics. *APL Bioeng.* 6, 036101. 10.1063/5.0092860 35818479 PMC9270995

[DEV202853C21] Givony, T., Leshkowitz, D., Del Castillo, D., Nevo, S., Kadouri, N., Dassa, B., Gruper, Y., Khalaila, R., Ben-Nun, O., Gome, T. et al. (2023). Thymic mimetic cells function beyond self-tolerance. *Nature* 622, 164-172. 10.1038/s41586-023-06512-8 37674082

[DEV202853C22] Gordon, J. and Manley, N. R. (2011). Mechanisms of thymus organogenesis and morphogenesis. *Development* 138, 3865-3878. 10.1242/dev.059998 21862553 PMC3160085

[DEV202853C23] Goyal, G., Prabhala, P., Mahajan, G., Bausk, B., Gilboa, T., Xie, L., Zhai, Y., Lazarovits, R., Mansour, A., Kim, M. S. et al. (2022). Ectopic lymphoid follicle formation and human seasonal influenza vaccination responses recapitulated in an organ-on-a-chip. *Adv. Sci.* 9, e2103241. 10.1002/advs.202103241 PMC9109055 35289122

[DEV202853C24] Hao, Y., Hao, S., Andersen-nissen, E., Gottardo, R., Smibert, P., Hao, Y., Hao, S., Andersen-nissen, E., Iii, W. M. M., Zheng, S. et al. (2021). Integrated analysis of multimodal single-cell data. *Cell* 184, 3573-3587.e29. 10.1016/j.cell.2021.04.048 34062119 PMC8238499

[DEV202853C25] Hofer, M. and Lutolf, M. P. (2021). Engineering organoids. *Nat. Rev. Mater.* 6, 402-420. 10.1038/s41578-021-00279-y 33623712 PMC7893133

[DEV202853C26] Howe, D. G., Blake, J. A., Bradford, Y. M., Bult, C. J., Calvi, B. R., Engel, S. R., Kadin, J. A., Kaufman, T. C., Kishore, R., Laulederkind, S. J. F. et al. (2018). Model organism data evolving in support of translational medicine. *Lab. Anim. (NY).* 47, 277-289. 10.1038/s41684-018-0150-4 30224793 PMC6322546

[DEV202853C27] Hun, M., Ramshaw, J., Chidgey, A. P., Barsanti, M., Wong, K. and Werkmeister, J. (2016). Native thymic extracellular matrix improves in vivo thymic organoid T cell output, and drives in vitro thymic epithelial cell differentiation. *Biomaterials* 118, 1-15. 10.1016/j.biomaterials.2016.11.054 27940379

[DEV202853C28] Irla, M., Hollander, G. and Reith, W. (2010). Control of central self-tolerance induction by autoreactive CD4+ thymocytes. *Trends Immunol.* 31, 71-79. 10.1016/j.it.2009.11.002 20004147

[DEV202853C29] James, K. D., Jenkinson, W. E. and Anderson, G. (2021). Non-Epithelial stromal cells in thymus development and function. *Front. Immunol.* 12, 634367. 10.3389/fimmu.2021.634367 33717173 PMC7946857

[DEV202853C30] Jenkinson, E. J., Anderson, G. and Owen, J. J. T. (1992). Studies on T cell maturation on defined thymic stromal cell populations in vitro. *J. Exp. Med.* 176, 845-853. 10.1084/jem.176.3.845 1512547 PMC2119352

[DEV202853C31] Kadouri, N., Nevo, S., Goldfarb, Y. and Abramson, J. (2020). Thymic epithelial cell heterogeneity: TEC by TEC. *Nat. Rev. Immunol.* 20, 239-253. 10.1038/s41577-019-0238-0 31804611

[DEV202853C32] Kernfeld, E. M., Genga, R. M. J., Neherin, K., Magaletta, M. E., Xu, P. and Maehr, R. (2018). A single-cell transcriptomic atlas of thymus organogenesis resolves cell types and developmental maturation. *Immunity* 48, 1258-1270.e6. 10.1016/j.immuni.2018.04.015 29884461 PMC6013397

[DEV202853C33] Kim, S., Shah, S. B., Graney, P. L. and Singh, A. (2019). Multiscale engineering of immune cells and lymphoid organs. *Nat. Rev. Mater.* 4, 355-378. 10.1038/s41578-019-0100-9 31903226 PMC6941786

[DEV202853C34] Klein, F., Veiga-Villauriz, C., Börsch, A., Maio, S., Palmer, S., Dhalla, F., Handel, A. E., Zuklys, S., Calvo-Asensio, I., Musette, L. et al. (2023). Combined multidimensional single-cell protein and RNA profiling dissects the cellular and functional heterogeneity of thymic epithelial cells. *Nat. Commun.* 14, 4071. 10.1038/s41467-023-39722-9 37429879 PMC10333192

[DEV202853C35] Lai, L. and Jin, J. (2009). Generation of thymic epithelial cell progenitors by mouse embryonic stem cells. *Stem Cells* 27, 3012-3020. 10.1002/stem.238 19824081

[DEV202853C36] Lai, A. Y. and Kondo, M. (2007). Identification of a bone marrow precursor of the earliest thymocytes in adult mouse. *Proc. Natl. Acad. Sci. USA* 104, 6311-6316. 10.1073/pnas.0609608104 17404232 PMC1851047

[DEV202853C37] Lavaert, M., Liang, K. L., Vandamme, N., Park, J. E., Roels, J., Kowalczyk, M. S., Li, B., Ashenberg, O., Tabaka, M., Dionne, D. et al. (2020). Integrated scRNA-seq identifies human postnatal thymus seeding progenitors and regulatory dynamics of differentiating immature thymocytes. *Immunity* 52, 1088-1104.e6. 10.1016/j.immuni.2020.03.019 32304633

[DEV202853C38] Lepletier, A., Hun, M. L., Hammett, M. V., Wong, K., Naeem, H., Hedger, M., Loveland, K. and Chidgey, A. P. (2019). Interplay between Follistatin, Activin A, and BMP4 signaling regulates postnatal thymic epithelial progenitor cell differentiation during aging. *Cell Rep.* 27, 3887-3901.e4. 10.1016/j.celrep.2019.05.045 31242421

[DEV202853C39] Liberzon, A., Birger, C., Thorvaldsdóttir, H., Ghandi, M., Mesirov, J. P. and Tamayo, P. (2015). The molecular signatures database hallmark gene set collection. *Cell Syst.* 1, 417-425. 10.1016/j.cels.2015.12.004 26771021 PMC4707969

[DEV202853C40] Lim, S., J. F. van Son, G., Wisma Eka Yanti, N. L., Andersson-Rolf, A., Willemsen, S., Korving, J., Lee, H. G., Begthel, H. and Clevers, H. (2024). Derivation of functional thymic epithelial organoid lines from adult murine thymus. *Cell Rep.* 43, 114019. 10.1016/j.celrep.2024.114019 38551965

[DEV202853C41] Luis, T. C., Luc, S., Mizukami, T., Boukarabila, H., Thongjuea, S., Woll, P. S., Azzoni, E., Giustacchini, A., Lutteropp, M., Bouriez-Jones, T. et al. (2016). Initial seeding of the embryonic thymus by immune-restricted lympho-myeloid progenitors. *Nat. Immunol.* 17, 1424-1435. 10.1038/ni.3576 27695000 PMC5172420

[DEV202853C42] Meireles, C., Ribeiro, A. R., Pinto, R. D., Leitão, C., Rodrigues, P. M. and Alves, N. L. (2017). Thymic crosstalk restrains the pool of cortical thymic epithelial cells with progenitor properties. *Eur. J. Immunol.* 47, 958-969. 10.1002/eji.201746922 28318017

[DEV202853C43] Michelson, D. A., Hase, K., Kaisho, T., Benoist, C., Michelson, D. A., Hase, K., Kaisho, T., Benoist, C. and Mathis, D. (2022). Thymic epithelial cells co-opt lineage-defining transcription factors to eliminate autoreactive T cells. *Cell* 185, 2542-2558.e18. 10.1016/j.cell.2022.05.018 35714609 PMC9469465

[DEV202853C44] Mingueneau, M., Kreslavsky, T., Gray, D., Heng, T., Cruse, R., Ericson, J., Bendall, S., Spitzer, M. H., Nolan, G. P., Kobayashi, K. et al. (2013). The transcriptional landscape of αβ T cell differentiation. *Nat. Immunol.* 14, 619-632. 10.1038/ni.2590 23644507 PMC3660436

[DEV202853C45] Mohtashami, M. and Zúñiga-Pflücker, J. C. (2006). Cutting edge: three-dimensional architecture of the thymus is required to maintain delta-like expression necessary for inducing T cell development. *J. Immunol.* 176, 730-734. 10.4049/jimmunol.176.2.730 16393955

[DEV202853C46] Montel-Hagen, A., Sun, V., Casero, D., Tsai, S., Zampieri, A., Jackson, N., Li, S., Lopez, S., Zhu, Y., Chick, B. et al. (2020). In vitro recapitulation of murine thymopoiesis from single hematopoietic stem cells. *Cell Rep.* 33, 108320. 10.1016/j.celrep.2020.108320 33113379 PMC7727762

[DEV202853C47] Nusser, A., Sagar, Swann, J. B., Krauth, B., Diekhoff, D., Calderon, L., Happe, C., Grün, D. and Boehm, T. (2022). Developmental dynamics of two bipotent thymic epithelial progenitor types. *Nature* 606, 165-171. 10.1038/s41586-022-04752-8 35614226 PMC9159946

[DEV202853C48] Owen, J. J. T. and Ritter, M. A. (1969). Tissue interaction in the development of thymus lymphocytes. *J. Exp. Med.* 129, 431-442. 10.1084/jem.129.2.431 5762051 PMC2138610

[DEV202853C49] Parent, A. V., Russ, H. A., Khan, I. S., Laflam, T. N., Metzger, T. C., Anderson, M. S. and Hebrok, M. (2013). Generation of functional thymic epithelium from human embryonic stem cells that supports host T cell development. *Cell Stem Cell* 13, 219-229. 10.1016/j.stem.2013.04.004 23684540 PMC3869399

[DEV202853C50] Park, J. E., Botting, R. A., Conde, C. D., Popescu, D. M., Lavaert, M., Kunz, D. J., Goh, I., Stephenson, E., Ragazzini, R., Tuck, E. et al. (2020). A cell atlas of human thymic development defines T cell repertoire formation. *Science* 367, eaay3224. 10.1126/science.aay3224 32079746 PMC7611066

[DEV202853C51] Poznansky, M. C., Evans, R. H., Foxall, R. B., Olszak, I. T., Piascik, A. H., Hartman, K. E., Brander, C., Meyer, T. H., Pykett, M. J., Chabner, K. T. et al. (2000). Efficient generation of human T cells from a tissue-engineered thymic organoid. *Nat. Biotechnol.* 18, 729-734. 10.1038/77288 10888839

[DEV202853C52] Purwada, A. and Singh, A. (2017). Immuno-engineered organoids for regulating the kinetics of B-cell development and antibody production. *Nat. Protoc.* 12, 168-182. 10.1038/nprot.2016.157 28005068 PMC6355337

[DEV202853C53] Ragazzini, R., Boeing, S., Zanieri, L., Green, M., D'Agostino, G., Bartolovic, K., Agua-Doce, A., Greco, M., Watson, S. A., Batsivari, A. et al. (2023). Defining the identity and the niches of epithelial stem cells with highly pleiotropic multilineage potency in the human thymus. *Dev. Cell* 58, 2428-2446.e9. 10.1016/j.devcel.2023.08.017 37652013 PMC10957394

[DEV202853C54] Ramos, S. A., Morton, J. J., Yadav, P., Reed, B., Alizadeh, S. I., Shilleh, A. H., Perrenoud, L., Jaggers, J., Kappler, J., Jimeno, A. et al. (2022). Generation of functional human thymic cells from induced pluripotent stem cells. *J. Allergy Clin. Immunol.* 149, 767-781.e6. 10.1016/j.jaci.2021.07.021 34331993 PMC8815270

[DEV202853C55] Reiner, A., Yekutieli, D. and Benjamini, Y. (2003). Identifying differentially expressed genes using false discovery rate controlling procedures. *Bioinformatics* 19, 368-375. 10.1093/bioinformatics/btf877 12584122

[DEV202853C56] Robinson, M. D., Mccarthy, D. J. and Smyth, G. K. (2010). edgeR : a Bioconductor package for differential expression analysis of digital gene expression data. *Bioinformatics* 26, 139-140. 10.1093/bioinformatics/btp616 19910308 PMC2796818

[DEV202853C57] Rossi, S. W., Jeker, L. T., Ueno, T., Kuse, S., Keller, M. P., Zuklys, S., Gudkov, A. V., Takahama, Y., Krenger, W., Blazar, B. R. et al. (2007). Keratinocyte growth factor (KGF) enhances postnatal T-cell development via enhancements in proliferation and function of thymic epithelial cells. *Blood* 109, 3803-3811. 10.1182/blood-2006-10-049767 17213286 PMC1874572

[DEV202853C58] Rossi, G., Manfrin, A. and Lutolf, M. P. (2018). Progress and potential in organoid research. *Nat. Rev. Genet.* 19, 671-687. 10.1038/s41576-018-0051-9 30228295

[DEV202853C59] Rothenberg, E. V. (2021). Single-cell insights into the hematopoietic generation of T-lymphocyte precursors in mouse and human. *Exp. Hematol.* 95, 1-12. 10.1016/j.exphem.2020.12.005 33454362 PMC8018899

[DEV202853C60] Seet, C. S., He, C., Bethune, M. T., Li, S., Chick, B., Gschweng, E. H., Zhu, Y., Kim, K., Kohn, D. B., Baltimore, D. et al. (2017). Generation of mature T cells from human hematopoietic stem and progenitor cells in artificial thymic organoids. *Nat. Methods* 14, 521-530. 10.1038/nmeth.4237 28369043 PMC5426913

[DEV202853C61] Sheridan, J. M., Taoudi, S., Medvinsky, A. and Blackburn, C. C. (2009). A novel method for the generation of reaggregated organotypic cultures that permits juxtaposition of defined cell populations. *Genesis* 351, 346-351. 10.1002/dvg.20505 19370754

[DEV202853C62] Smyth, G. K., Ritchie, M. E., Law, C. W., Alhamdoosh, M., Su, S., Dong, X. and Tian, L. (2018). RNA-seq analysis is easy as 1-2-3 with limma, Glimma and edgeR. *F1000Res.* 5, 1408. 10.12688/f1000research.9005.3 PMC4937821 27441086

[DEV202853C63] Steier, Z., Aylard, D. A., McIntyre, L. L., Baldwin, I., Jeong Yoon Kim, E., Lutes, L. K., Ergen, C., Huang, T.-S., Robey, E. A., Yosef, N. et al. (2023). Single-cell multi-omic analysis of thymocyte development reveals drivers of CD4/CD8 lineage commitment. *Nat. Immunol.* 24, 1579-1590. 10.1038/s41590-023-01584-0 37580604 PMC10457207

[DEV202853C64] Subramanian, A., Tamayo, P., Mootha, V. K., Mukherjee, S., Ebert, B. L., Gillette, M. A., Paulovich, A., Pomeroy, S. L., Golub, T. R., Lander, E. S. et al. (2005). Gene set enrichment analysis: a knowledge-based approach for interpreting genome-wide expression profiles. *Proc. Natl. Acad. Sci. USA* 102, 15545-15550. 10.1073/pnas.0506580102 16199517 PMC1239896

[DEV202853C65] Sun, X., Xu, J., Lu, H., Liu, W., Miao, Z., Sui, X., Liu, H., Su, L., Du, W., He, Q. et al. (2013). Directed differentiation of human embryonic stem cells into thymic epithelial progenitor-like cells reconstitutes the thymic microenvironment in vivo. *Cell Stem Cell* 13, 230-236. 10.1016/j.stem.2013.06.014 23910085

[DEV202853C66] Wagar, L. E., Salahudeen, A., Constantz, C. M., Wendel, B. S., Lyons, M. M., Mallajosyula, V., Jatt, L. P., Adamska, J. Z., Blum, L. K., Gupta, N. et al. (2021). Modeling human adaptive immune responses with tonsil organoids. *Nat. Med.* 27, 125-135. 10.1038/s41591-020-01145-0 33432170 PMC7891554

[DEV202853C67] Wong, K., Lister, N. L., Barsanti, M., Lim, J. M. C., Hammett, M. V., Khong, D. M., Siatskas, C., Gray, D. H. D., Boyd, R. L. and Chidgey, A. P. (2014). Multilineage potential and self-renewal define an epithelial progenitor cell population in the adult thymus. *Cell Rep.* 8, 1198-1209. 10.1016/j.celrep.2014.07.029 25131206

[DEV202853C68] Zhou, W., Yui, M. A., Williams, B. A., Yun, J., Wold, B. J., Cai, L. and Rothenberg, E. V. (2019). Single-cell analysis reveals regulatory gene expression dynamics leading to lineage commitment in early T cell development. *Cell Syst.* 9, 321-337.e9. 10.1016/j.cels.2019.09.008 31629685 PMC6932747

[DEV202853C69] Žuklys, S., Handel, A., Zhanybekova, S., Govani, F., Keller, M., Maio, S., Mayer, C. E., Ying Teh, H., Hafen, K., Gallone, G. et al. (2016). Foxn1 regulates key target genes essential for T cell development in postnatal thymic epithelial cells. *Nat. Immunol.* 17, 1206-1215. 10.1038/ni.3537 27548434 PMC5033077

