# Supplementary material for: Thymic epithelial organoids mediate T-cell development
Source: Development. 2024 Sep 3;151(17):dev202853. doi: 10.1242/dev.202853 (PMC11441983; doi:10.1242/dev.202853)
Supplement: Supplementary information [file develop-151-202853-s1.pdf]

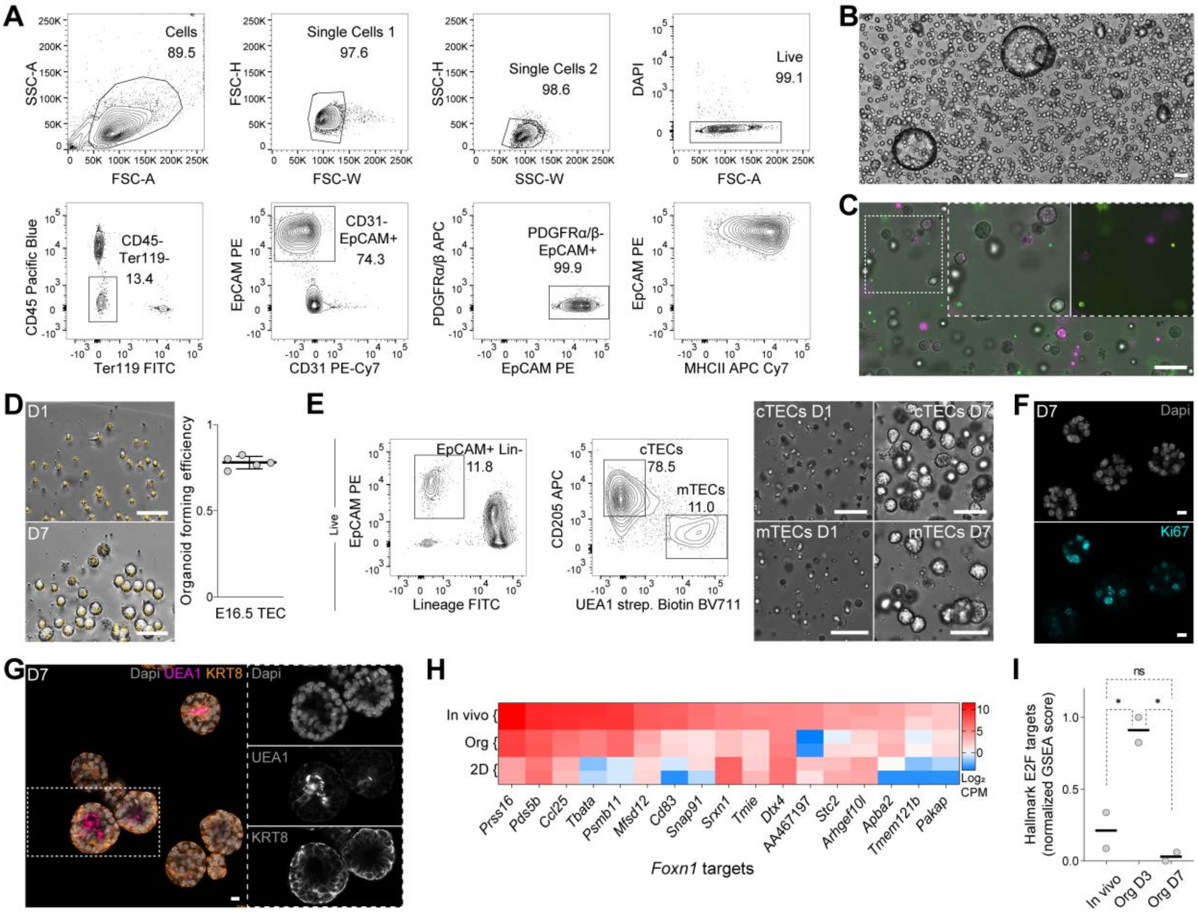

Fig. S1. Establishment of thymic epithelial organoids. (**A**) Sorting strategy to isolate TECs (EpCAM+). Exclusion of CD45+, Ter119+, CD31+ and PDGFRα+ PDGFRβ+ cells. TECs are mostly MHCII+. (**B**) Thymic epithelial organoids can also be established from neonatal (P0) TECs. (**C**) Single-coloured D7 TEC organoids originating from sorted TECs labelled with CellTrace dyes. Boxed area is shown at higher magnification and with only fluorescent channels. (**D**) Organoid-forming efficiency calculation by comparing the same field of view at D1 and D7. (**E**) cTEC- and mTEC-derived organoids. Left: Flow cytometry plots for isolating cTECs and mTECs. Gating strategy is indicated on the left. Lin: Lineage. Strep: streptavidin. Right: Both populations cultured separately have the potential to form organoids. (**F, G**) Immunofluorescence images of TEC organoids. (F) D7 organoids contain both proliferating (Ki67 [cyan], bottom) and non-proliferating cells. (G) Different cell populations are present in D7 organoids, here with medullary cells (UEA1-reactivity [bright pink and middle] and cortical cells (KRT8 [amber and bottom]). Dapi (grey) stains nuclei. Insert shows the 3 channels of the boxed area separately. (**H**)

Development • Supplementary information

Heatmap displaying *Foxn1* target gene expression for freshly extracted TECs (*In vivo*), thymic epithelial organoids (Org) and TECs cultured in 2D (2D). n = 2 mice per condition. (**I**) Single-sample gene set enrichment analysis (GSEA) demonstrating a proliferation peak (E2F hallmark) for D3 organoids. *$P=0.0241$ (*In vivo* vs Org. D3) and *$P=0.0126$ (Org D3 vs Org D7), ns, not significant ($P>0.05$) (one-way ANOVA with Tukey's multiple comparison test; n = 2 mice per condition). Graph represents individual datapoints with mean. All TECs originate from E16.5 thymi except in B. Scale bars: 100 µm (B -E), 10 µm (F,G) .

Development • Supplementary information

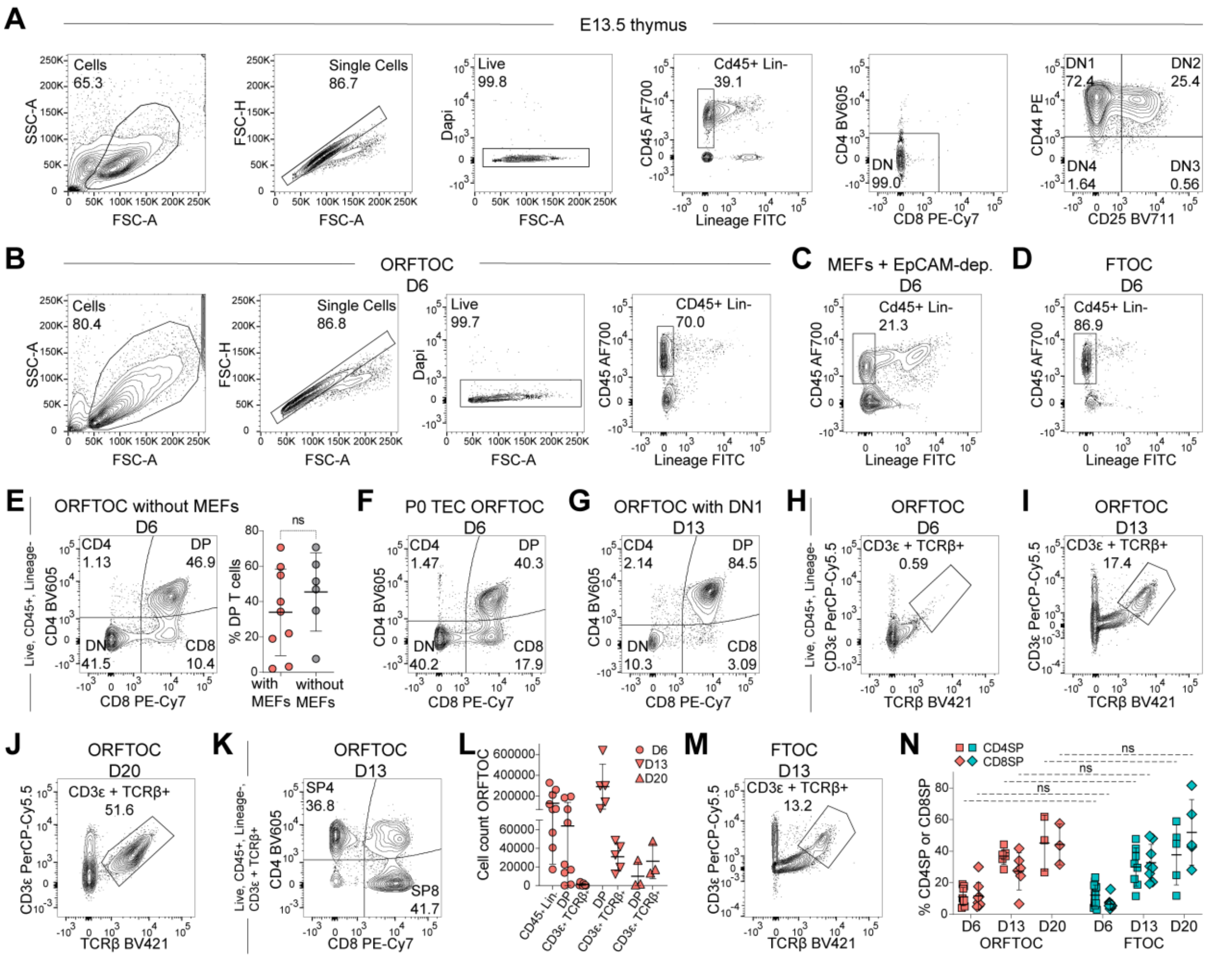

Fig. S2. Generation and analysis of ORFTOCs and control conditions. (A-N) Flow cytometry plots and respective quantifications. (A) Thymocytes in E13.5 thymi are at the DN stage. (B) Gating strategy used to analyse thymocyte development in ORFTOCs and control conditions. The last plot highlights the CD45+ Lineage- (Lin-) population in ORFTOCs at D6. (C, D) CD45+ Lin- population at D6 in control reaggregates with MEFs and the EpCAM-depleted fraction of cells from E13.5 thymi (C) and in FTOC controls (D). (E) Thymocyte development in ORFTOCs without MEFs at D6 and comparison between ORFTOCs with and without MEFs. ns, not significant (*P=0.3744*) (unpaired t-test, n = 9 and 6 for ORFTOCs with MEFs and without, respectively, from at least 4 different experiments). (F) Thymocyte development in ORFTOCs composed of TECs from organoids derived from P0 mice. (G) Thymocyte development in D13 ORFTOCs made with adult DN1 as T-cell input population. (H-J) CD3ε+ TCRβ+ population in D6 (H), D13 (I) and D20 (J) ORFTOCs. (K) Division of the CD3ε+ TCRβ+ in D13 ORFTOCs into CD4SP and CD8SP T-cell lineages. (L) Cell count for the main populations in ORFTOCs at D6, D13 and D20. (M) CD3ε+ TCRβ+ population in D13 FTOC controls. (N) Percentage of CD4SP and

Development • Supplementary information

CD8SP within the CD3ε+ TCRβ+ population, at D6, D13 and D20 for both ORFTOCs and FTOC controls. ns, not significant (*P>0.05*) (Kruskal-Wallis test for CD4SP and for CD8SP with Dunn's multiple comparisons test; n = 8, 5, 3 for ORFTOC CD4SP and CD8SP populations at D6, D13 and D20 respectively, and n = 14, 8 ,5 for FTOC CD4SP and CD8SP populations at D6, D13 and D20 respectively, from at least 3 independent experiments). ORFTOCs were formed with TECs cultured as organoids originating from E16.5 thymi, with the EpCAM-depleted fraction of cells from E13.5 thymi and with MEFs except where otherwise specified. FTOCs were from E13.5 thymi. Where needed, gating strategies are indicated on the left of the first flow cytometry plot displaying each population. Quantification graphs represent individual datapoints with mean±s.d.

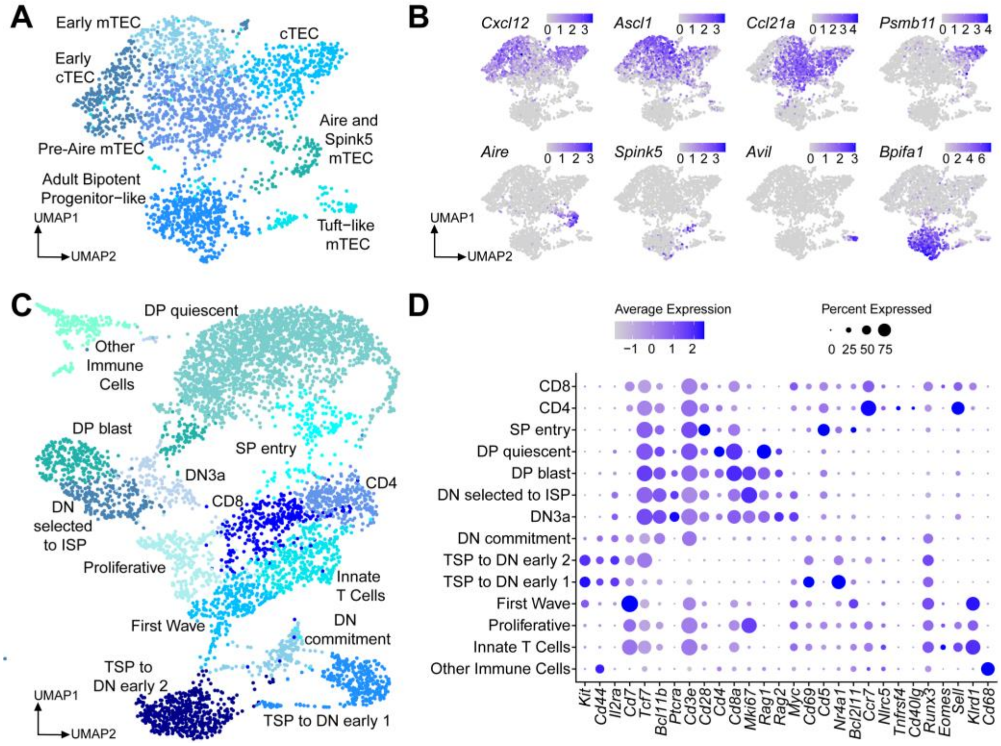

Fig. S3. Single-cell transcriptomic characterization of ORFTOCs and FTOCs. (**A-C**) UMAPs. (A) Epithelial clusters. (B) Characteristic marker expression for each of the epithelial clusters. (C) Immune clusters. (**D**) Dot plot summarizing the expression of characteristic markers of T-cell development for the different clusters. ORFTOCs were formed with TECs cultured as organoids originating from E16.5 thymi, with the EpCAM-depleted fraction of cells from E13.5 thymi and with MEFs. FTOCs were from E13.5 thymi.

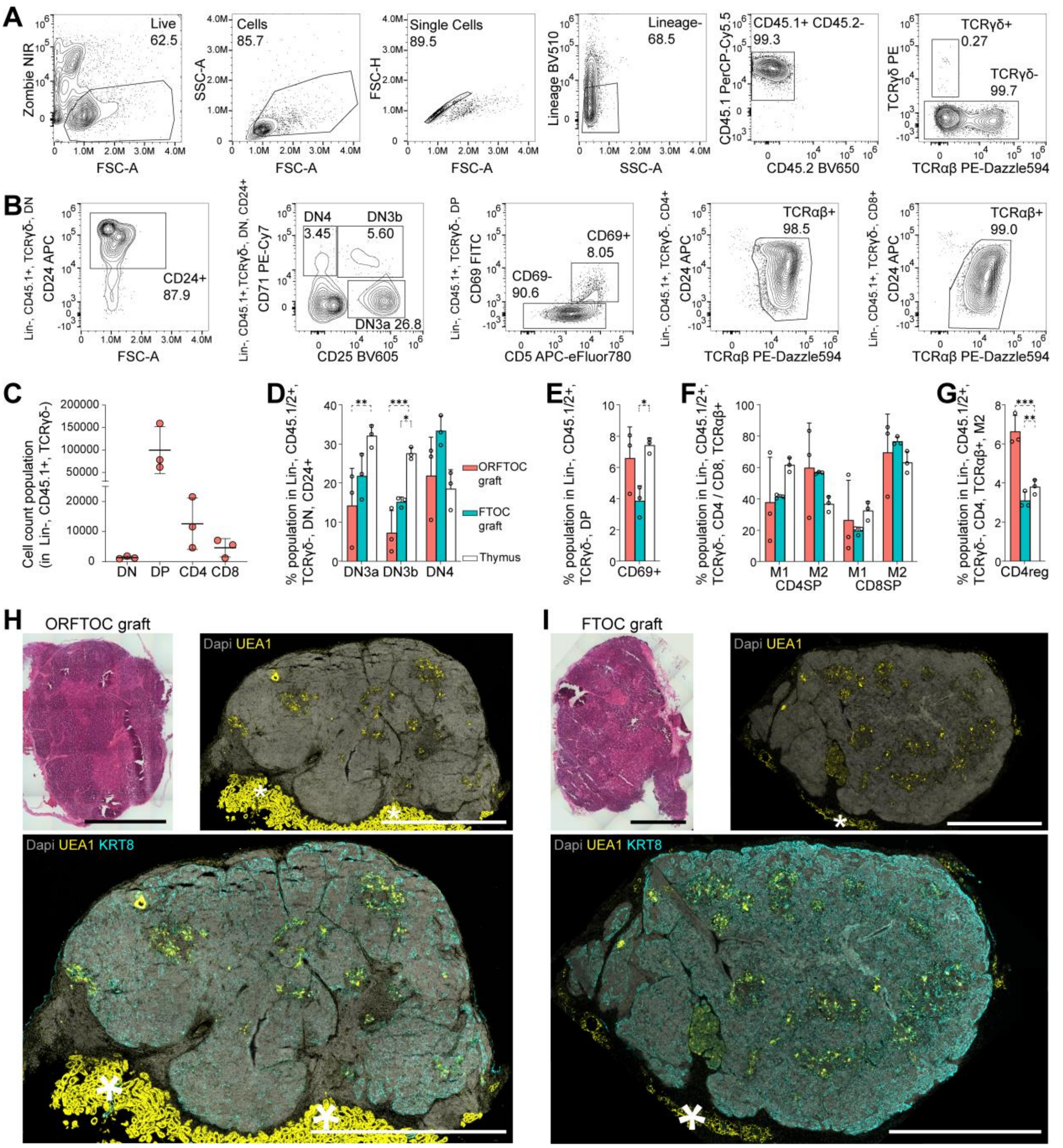

Fig. S4. Analysis of ORFTOC grafts and comparison to controls. (**A-F**) Flow cytometry plots and respective quantifications. ORFTOCs (CD45.2) were grafted under the kidney capsule of CD45.1 hosts. Live, single, Lineage negative (Lin-) cells were gated for CD45.1 positivity (A) and TCRγδ negative T cells were further analysed (B). (C) Cell count for the main populations (DN, DP, CD4 and CD8) in ORFTOC grafts, related to Fig. 4D. CD25 and CD71 expression on CD24+ DN cells were used to enumerate DN3a, DN3b and DN4 subsets, which are quantified in (D) for the different conditions. β-selection occurs at the DN3a to DN3b transition. CD69 expression in DP is quantified in (E) for the different condition, and identifies cells undergoing positive selection. Gating on the TCRαβ+ population, mature (M1 [CD24+

CD69+] and M2 [CD24+ CD69-]) CD4+ and CD8+ SP thymocytes are quantified in (F) for the different conditions. Within the M2 population, CD4 regulatory T cells (CD4reg, CD25+) are quantified in (G) for the different conditions. For all bar graphs, only significant differences (*P≤0.05*) are indicated with stars. **P=0.0381* (DN3a ORFTOC grafts vs thymi), ****P=0.0009* (DN3b ORFTOC grafts vs thymi), **P=0.0116* (DN3b FTOC grafts vs thymi), **P=0.0373* (CD69+ FTOC grafts vs thymi), (one-way ANOVA with Tukey's multiple comparison test for each subpopulation between conditions, n = 3 grafts/mice for each condition). Bar graphs represent mean±s.d., with individual datapoints displayed as circles. (**H, I**) Haematoxylin and eosin (H&E) staining and immunostaining for UEA1 (yellow) and KRT8 (cyan) in ORFTOC and FTOC grafts. Dapi (grey) stains nuclei. Asterisks indicate kidney tissue surrounding grafts. ORFTOCs were formed with TECs cultured as organoids originating from E16.5 thymi, with the EpCAM-depleted fraction of cells from E13.5 thymi and with MEFs. FTOCs were from E13.5 thymi. Scale bars: 1000 μm.

Table S1,2. Differentially expressed genes and Voom expression from bulk RNA sequencing analysis. Tables with differential expression and Voom expression for the three conditions (*In vivo*, Organoids and 2D) analysed in the article.

Available for download at
https://journals.biologists.com/dev/article-lookup/doi/10.1242/dev.202853#supplementary-data

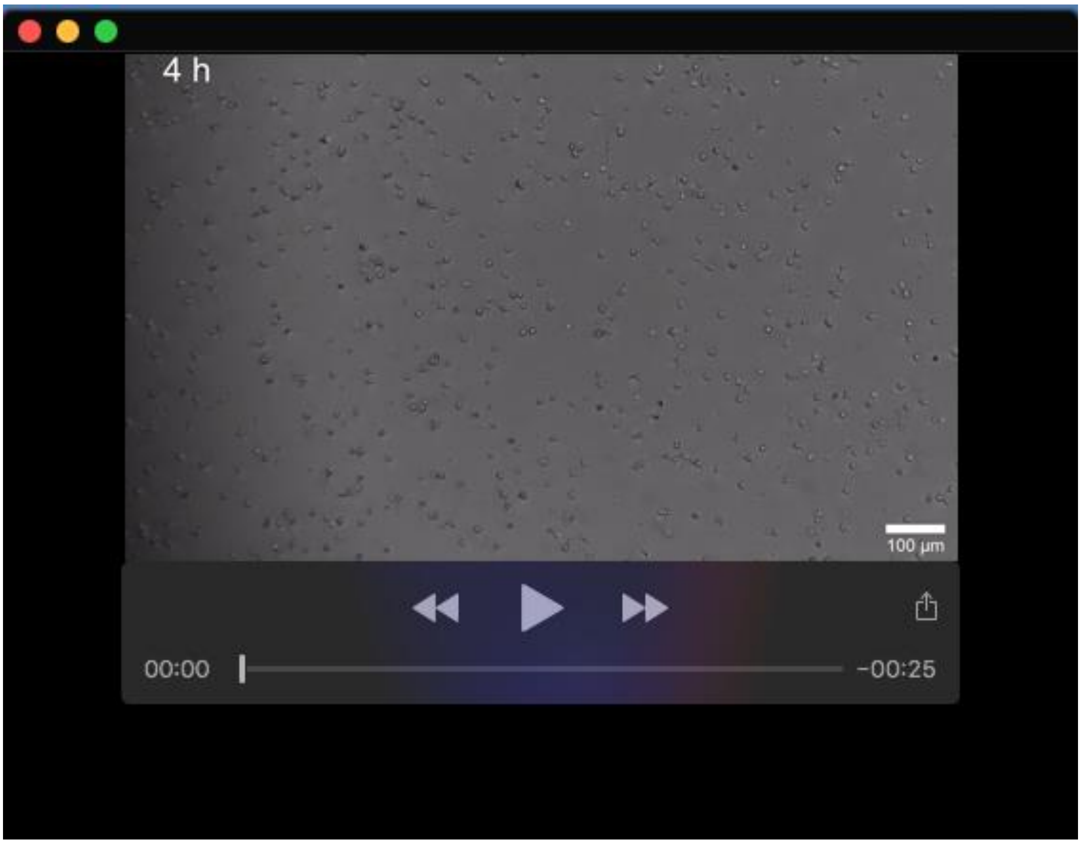

Movie 1. Thymic epithelial organoid establishment. One-week time-lapse video showing the development of thymic epithelial organoids starting from sorted single E16.5 TECs seeded in Matrigel and cultured in defined conditions.

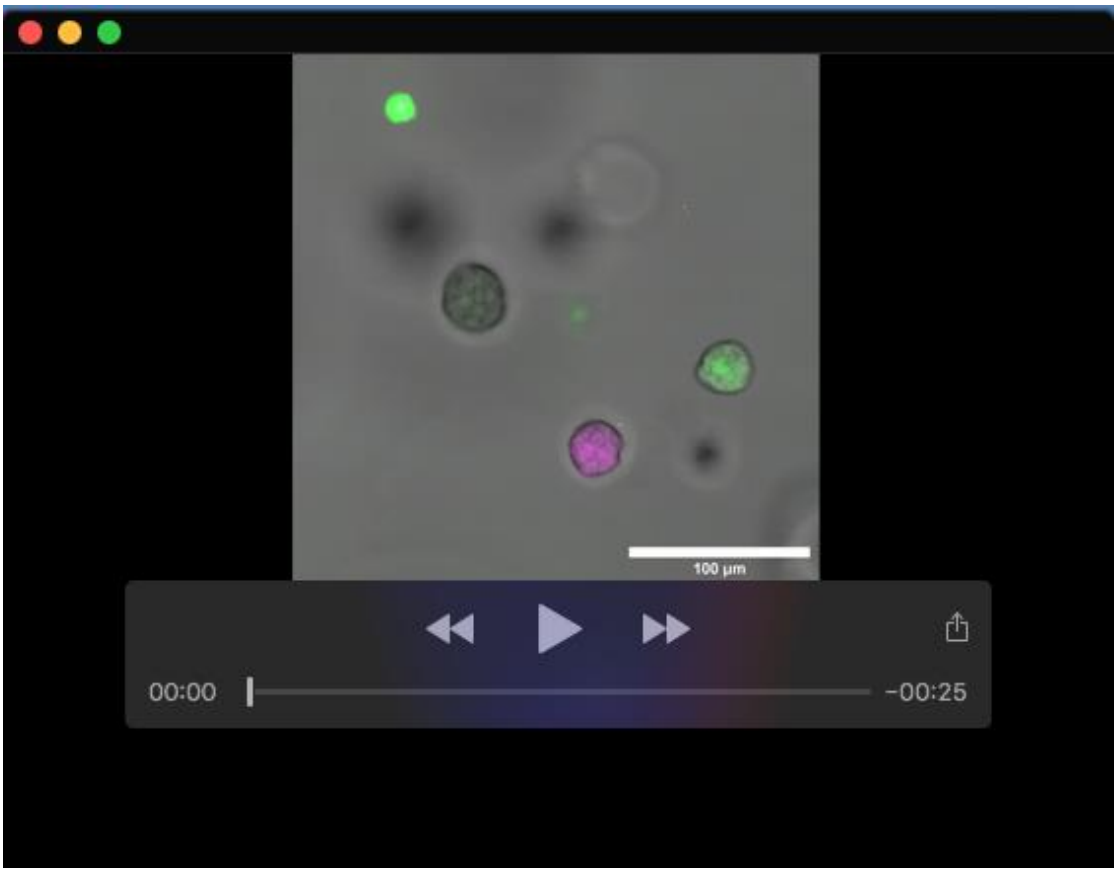

Movie 2. Single-coloured thymic epithelial organoids. Z-stack of thymic epithelial organoids at day 7 that grew from single E16.5 sorted TECs labelled with either CellTrace Oregon Green 488 (carboxy-DFFDA SE) or CellTrace Far Red and seeded at a 1:1 ratio in Matrigel.
