# Supplementary material for: Thymic epithelial organoids mediate T-cell development
Source: Development. 2024 Sep 3;151(17):dev202853. doi: 10.1242/dev.202853 (PMC11441983; doi:10.1242/dev.202853)
Supplement: Supplementary file 1 [file develop-151-202853_review_history.pdf]

# Thymic epithelial organoids mediate T-cell development

Tania L. Hübscher, L. Francisco Lorenzo-Martiń, Thomas Barthlott, Lucie Tillard, Jakob J. Langer, Paul Rouse, C. Clare Blackburn, Georg Holländer and Matthias P. Lutolf
DOI: 10.1242/dev.202853

**Editor**: Hanna Mikkola

**Review timeline**
Original submission: 5 March 2024
Editorial decision: 23 April 2024
First revision received: 13 June 2024
Accepted: 9 July 2024

---

**Original submission**

First decision letter

MS ID#: DEVELOP/2024/202853

MS TITLE: Thymic epithelial organoids mediate T cell development

AUTHORS: Tania Hubscher, Francisco Lorenzo-Martin, Thomas Barthlott, Lucie Tillard, Jakob Langer, Paul Rouse, Clare Blackburn, Georg Clare Hollander, and Matthias Lutolf

I have now received all the referees' reports on the above manuscript, and have reached a decision. The referees' comments are appended below, or you can access them online: please go to BenchPress and click on the 'Manuscripts with Decisions' queue in the Author Area.

As you will see, the referees express considerable interest in your work, but have some significant criticisms and recommend a substantial revision of your manuscript before we can consider publication. If you are able to revise the manuscript along the lines suggested, which may involve further experiments, I will be happy receive a revised version of the manuscript. Your revised paper will be re-reviewed by one or more of the original referees, and acceptance of your manuscript will depend on your addressing satisfactorily the reviewers' major concerns. Please also note that Development will normally permit only one round of major revision. If it would be helpful, you are welcome to contact us to discuss your revision in greater detail. Please send us a point-by-point response indicating your plans for addressing the referees' comments, and we will look over this and provide further guidance.

Please attend to all of the reviewers' comments and ensure that you clearly highlight all changes made in the revised manuscript. Please avoid using 'Tracked changes' in Word files as these are lost in PDF conversion. I should be grateful if you would also provide a point-by-point response detailing how you have dealt with the points raised by the reviewers in the 'Response to Reviewers' box. If you do not agree with any of their criticisms or suggestions please explain clearly why this is so.

Reviewer 1

*Advance summary and potential significance to field*

The study by Hubscher et al. presents a novel method to prospectively isolate and expand thymic epithelial cell progenitors (TEPs) from the embryonic thymus, with the aim of constructing thymic

© 2024. Published by The Company of Biologists under the terms of the Creative Commons Attribution License (https://creativecommons.org/licenses/by/4.0/). 

epithelial organoids. This topic is of great interest and relevance in the thymus field given the current challenges in developing thymic organoids that fully mimic endogenous thymus activity for their potential integration into long-term thymic replacement strategies. The study is both interesting and well-conducted, with extensive characterization of thymic epithelial organoids and their lineage and functional contributions to thymopoiesis. The authors begin by demonstrating that TECs from e16.5 embryonic thymus can form clonal-derived thymic epithelial organoids, which closely mimic the transcriptional profile of ex vivo isolated TECs, particularly for a panel of selected genes (Fig 1). They further show, through the use of RTOCs established with TECs from organoid cultures, that these organoids are capable of contributing to thymopoiesis in a chimeric RTOC where they serve as the sole source of TEC (Fig 2). Additionally, using scRNA sequencing they show that the progeny of organoid-derived TECs contribute to various clusters known to exist within the endogenous thymus, at least to a certain extent (Fig 3). Finally, RTOCs containing organoid-derived TECs are shown to sustain long-term thymopoiesis in vivo (Fig 4). While the experiments are largely well-done and the data thoroughly analysed, this reviewer considers that there are some points and shortcomings that the authors must address to enhance the scope of the study.

*Comments for the author*

1 – The introduction and discussion are indeed very succinct. If space permits the authors may benefit from briefly defining the current state of the art regarding the various methods used to prospectively isolate TEC progenitors from the ex vivo thymus or to expand them in vitro. They could cite some previous studies (PMID: 25131206, 26997270, 28318017) to provide context for a general reader. Additionally, it may be valuable to highlight the shortcomings of those assays in terms of the functional contribution of the selected TEC progenitors to T cell development, as demonstrated in this paper.
Furthermore, the authors may consider covering the fact that scRNAseq analysis has revealed a further degree of heterogeneity and dynamics among TEC progenitors
(including work from authors in the study or other studies, PMIDs: 32840480 35614226). This heterogeneity remains challenging to prospectively isolate due to the absence of definitive markers. This addition could provide a broader understanding of the current landscape of TEC research and underscore the significance of the novel methodology proposed in this study.

2 - The authors primarily tested the capacity of TECs from E16.5 embryonic thymi to form organoids. However, it is essential to consider (or discussed) whether this methodology can also isolate potential TEPs from early postnatal and/or adult thymi. If this has not been tested, it should be at least discussed in the study that the protocol applies in its current state to the embryonic thymus. Addressing this point would provide clarity on the applicability of the methodology across different developmental stages of the thymus. Additionally, discussing the implications of applying this protocol to postnatal or adult thymi could help contextualize the findings for this rapidly evolving research area.

3 – What is the efficiency of organoid formation among sorted TECs? At E16.5 there are already some differentiated cortical and medullary TEC, including Aire-positive cells. Which cells are responsible for giving rise to the TEC organoids?

It appears that the authors sorted based on pan-EpCAM-positive total TECs.
4- Although data presented in figure 1, and supplementary movie, appears to suggest clonal origin for the TEC-derived organoids, have the authors conducted similar experiments using limiting dilution of TEC numbers or single-cell sorting?
Alternatively, mixed TECs from reporter mice with distinct fluorescence markers (e.g., RFP vs YFP) could confirm clonality? In this latter system, clonal organoids should be monocromatic, providing more solid evidence of their clonal origin.

5 – The genome-wide RNA sequence analysis depicted in Fig 1G seems selective and could be extended to include a larger-scale comparison of differentially expressed genes (DEGs) among ex vivo, organoid, or 2D-derived TECs. This expanded analysis should also include a comprehensive comparison of Foxn1 targets (PMID: 27548434).
Specifically, do organoid-derived TECs match ex vivo isolated TECs from E16.5 thymus in terms of the full breadth of Foxn1 targets? This more broad comparison would provide valuable insights into

© 2024. Published by The Company of Biologists under the terms of the Creative Commons Attribution License (https://creativecommons.org/licenses/by/4.0/). 

the fidelity of organoid-derived TECs to mimic the transcriptional profile of their ex vivo counterparts.

6 – The lineage and functional potential assays set in Figures 2-4 appears to be conducted with a pool of TEC-derived organoids. In this system, it is possible that only a fraction of organoids contribute to TEC lineage and/or may contribute to different TEC lineages, such as cortical and medullary subsets. Despite possible phenotypic similarities as shown in Figure 1, possible inter-clonal heterogeneity may determine different lineage and functional potentials. Can the authors comment (and discuss) on this aspect?

7- The lineage and functional potential assays depicted in Figures 2-4 seem to have been conducted using a pool of TEC-derived organoids. In this setup, it is plausible that only a subset of organoids contributes to the TEC lineage and/or may contribute to different TEC lineages, such as cortical and medullary subsets.
Despite potential phenotypic similarities as shown in Figure 1, the presence of inter-clonal heterogeneity could influence distinct lineage and functional potentials.
Commenting and discussing this aspect may provide a more balanced understanding of the results and their implications. Additionally, discussing strategies to mitigate possible heterogeneity in future experiments could strengthen the study's conclusions.

Minor point:
Sentence on lines 53-55 might need some revision. It seems to lack clarity or completeness after "which involve organotypic three-dimensional (3D) cultures containing different cell types."

Reviewer 2

*Advance summary and potential significance to field*

Thymic epithelial cells are a notoriously difficult cell type to successfully culture. With a severely limited number of successful in-vitro thymic organoid systems reported, there is a great need for new methods to successfully support T cell development in culture. With the overall decline in thymic function with age as a consequence of thymic involution, plus many clinical settings where thymic function is either absent or becomes compromised, the need for the development of successful thymic organoid cultures is of high importance, both clinically and to better understand the fundamentals of thymus biology. Hubscher et al aim to conquer the long-standing challenge of generating functional thymic organ cultures, by applying their expert knowledge of organoid systems used for other endoderm-derived organs. Authors clearly demonstrate they have developed a novel thymic organoid protocol, creating 3D thymic structures from embryonic TEC that are able to support the major stages of thymocyte development when reaggregated with T cell precursors. Moreover, when transplanted under the kidney of wildtype mice, the thymic organoids support normal T cell development of host derived T cell precursors. Overall, demonstrating the successful generation of a novel thymic organoid system.

*Comments for the author*

The manuscript is well written and easy to follow, and the data represents well designed and executed experiments. However, before the acceptance for publication in the Journal of Development, I recommend a number of changes to the manuscript. My most serious concerns are the absence of references to already established thymic organoid systems and the lack of clarity on how the culturing of E16.5 TEC first in the thymic organoid and then reaggregating in ORFTOCs offers an advantage over isolating TEC E16.5 from FTOC (with or without deoxyguanosine treatment) and then using these TEC in the pre-existing RTOC system.

There are additional changes I believe need to be made to the manuscript before it should be considered for publication.

The introduction is too short and brief. There is no clear explanation of what has previously been published in the thymic organoid field, nor has any explanation been given to the importance or

© 2024. Published by The Company of Biologists under the terms of the Creative Commons Attribution License (https://creativecommons.org/licenses/by/4.0/). 

clinical significance of successfully replicating T cell development in a thymic organoid system. A major expansion of the introduction is essential to highlight the novelty and impact of the work presented. Moreover, the abstract would also be strengthened if amended to include these changes. More justification is also needed to strengthen the logic of why applying approaches used for other endoderm-derived organs would improve pre-existing thymic culture systems. Additionally, no description is provided of how this approach is different compared to non-endoderm-derived organoids.

No justification is provided for why embryonic day E16.5 has been chosen. Could this method be applied to younger and older TEC sources. Would it work using NB and adult TEC? If so, this would clearly highlight the advantage of the thymic organoid system over traditional FTOC to RTOC methods. Moreover, authors suggest this organoid system may be able to maintain long term functionality and data is presented up to 13 days in culture. Can this culture system remain functional for over 13 days, highlighting an additional advantage of the thymic organoid system over traditional FTOC to RTOC methods.

Mouse embryonic fibroblasts (MEFs) were added to the reaggregates. How essential is this step. Would the organoids work without the addition of MEFs? The inclusion of a without MEF control group would address this issue.

The findings of the manuscript would be strengthened by the extension of the bulk RNA seq data analysis displayed in Fig. 1J. Is EpCAM expressed? What is the expression of mTEC markers such as CCL21a and Aire?
Do you see TRA expression? Aire dependent and Aire independent TRA?

The direct and indirect effects of FGF7 are unclear. Does the increase proliferation drive the changes in metabolism or vice versa. This relationship could be discussed in the text.

What are the numbers obtained from organoids? Can counts be provided? What numbers of TEC are put into the ORFTOCs and do they increase in size over the culture period? Is this at a comparable rate as the FTOC controls?

It is unclear if authors can disaggregate the ORFTOCs and stained for TEC markers by flow cytometry post culture, or this can only be done after grafting (Fig. S4). Moreover, in Figure 3D it is unclear how many ORFTOC were sorted for the scRNA and if several ORFTOC were pooled?

FTOC controls for scRNAseq were E13.5, but the TEC from ORFTOCs originated from E16.5 in the thymic organoids, therefore could this explain some of the differences observed. It also needs to be made clearer the age of the reference populations from Park et al. reference atlas (Fig. 3, E and F).

ORFTOC were transplanted under the kidney capsule of syngeneic CD45.1 recipient mice and harvest 5 weeks later. The data demonstrated the grafts were able to support the major stages of T cell development (DN/DP/SP4/SP8 subsets, and Treg). Were a population of CD45.1+ ORFTOC derived T cells detectable in the periphery of the recipients/hosts? This would suggest the T cells made in vitro were functional and could contribute to the peripheral pool of T cells.

Representative immunostaining confirming medullary and cortical regions are not very clear in figure 4G. Can images of a whole section of the graft be displayed that allows the overall assessment of proportion of medullary to cortical areas? TEC markers could be shown, but also CD4 and CD8 staining would reveal DP enriched cortical regions vs SP4/SP8 enriched medullary regions.

Are the T cells that are generated in the ORFTOC tolerant? Have the ORFTOC been grafted under the kidney capsule of nude mice and if so, do hosts show signs of autoimmunity? This would be an insightful experiment but is not essential for publication.

---

**First revision**

© 2024. Published by The Company of Biologists under the terms of the Creative Commons Attribution License (https://creativecommons.org/licenses/by/4.0/). 

Author response to reviewers' comments

**Reviewer 1** Advance Summary and Potential Significance to Field:
The study by Hübscher et al. presents a novel method to prospectively isolate and expand thymic epithelial cell progenitors (TEPs) from the embryonic thymus, with the aim of constructing thymic epithelial organoids. This topic is of great interest and relevance in the thymus field given the current challenges in developing thymic organoids that fully mimic endogenous thymus activity for their potential integration into long-term thymic replacement strategies. The study is both interesting and well-conducted, with extensive characterization of thymic epithelial organoids and their lineage and functional contributions to thymopoiesis. The authors begin by demonstrating that TECs from e16.5 embryonic thymus can form clonal-derived thymic epithelial organoids, which closely mimic the transcriptional profile of ex vivo isolated TECs, particularly for a panel of selected genes (Fig 1). They further show, through the use of RTOCs established with TECs from organoid cultures, that these organoids are capable of contributing to thymopoiesis in a chimeric RTOC where they serve as the sole source of TEC (Fig 2). Additionally, using scRNA sequencing they show that the progeny of organoid-derived TECs contribute to various clusters known to exist within the endogenous thymus, at least to a certain extent (Fig 3). Finally, RTOCs containing organoid-derived TECs are shown to sustain long-term thymopoiesis in vivo (Fig 4). While the experiments are largely well-done and the data thoroughly analysed, this reviewer considers that there are some points and shortcomings that the authors must address to enhance the scope of the study.
We thank the reviewer for the supportive comments on our work.

**Reviewer 1** Comments for the Author:
1 – The introduction and discussion are indeed very succinct. If space permits, the authors may benefit from briefly defining the current state of the art regarding the various methods used to prospectively isolate TEC progenitors from the ex vivo thymus or to expand them in vitro. They could cite some previous studies (PMID: 25131206, 26997270, 28318017) to provide context for a general reader. Additionally, it may be valuable to highlight the shortcomings of those assays in terms of the functional contribution of the selected TEC progenitors to T cell development, as demonstrated in this paper.
Furthermore, the authors may consider covering the fact that scRNAseq analysis has revealed a further degree of heterogeneity and dynamics among TEC progenitors (including work from authors in the study or other studies, PMIDs: 32840480, 35614226). This heterogeneity remains challenging to prospectively isolate due to the absence of definitive markers. This addition could provide a broader understanding of the current landscape of TEC research and underscore the significance of the novel methodology proposed in this study.
We agree with the reviewer comments. We initially aimed for a Research Report but the revised version has been updated to a Research Article, which left space for extending the introduction and discussion. We included previous studies suggested by the reviewer and mentioned their shortcomings to provide context for the current work. We thank the reviewer for their suggestion to elaborate on TEC progenitors identified by scRNAseq. These studies are now included in the discussion section, and we describe how thymic epithelial organoids might help shedding light on their potential to generate different TEC populations.

2 - The authors primarily tested the capacity of TECs from E16.5 embryonic thymi to form organoids. However, it is essential to consider (or discussed) whether this methodology can also isolate potential TEPs from early postnatal and/or adult thymi. If this has not been tested, it should be at least discussed in the study that the protocol applies in its current state to the embryonic thymus. Addressing this point would provide clarity on the applicability of the methodology across different developmental stages of the thymus. Additionally, discussing the implications of applying this protocol to postnatal or adult thymi could help contextualize the findings for this rapidly evolving research area.
We thank the reviewer for this insightful remark. We included data showing the possibility to generate functional thymic epithelial organoids from early postnatal TEPs (Fig. S2F). At its current stage, our protocol does not however lead to organoid formation when applied to sorted adult (4-week-old) TECs. For transparency, this is now included in the discussion.

3 – What is the efficiency of organoid formation among sorted TECs? At E16.5, there are already some differentiated cortical and medullary TEC, including Aire-positive cells. Which cells are

© 2024. Published by The Company of Biologists under the terms of the Creative Commons Attribution License (https://creativecommons.org/licenses/by/4.0/).

responsible for giving rise to the TEC organoids? It appears that the authors sorted based on pan-EpCAM-positive total TECs.

We aimed at answering this point in two ways. First, we estimated the efficiency of organoid formation from sorted total TECs (EpCAM+). We counted cells one day after sorting and organoids seven days after sorting on the same fields of view, across several experiments (Fig. S1D). The estimated organoid forming efficiency is 78 $\pm$ 3 % and now indicated in the main text. Second, as rightfully pointed out by the reviewer, the thymus at E16.5 already contain differentiated cortical and medullary TECs. We thus performed experiments where cTECs (EpCAM+ CD205+ UEA1-) and mTECs (EpCAM+ CD205- UEA1+) were sorted separately and showed that both can form organoids in our culture conditions (Fig. S1E). Future work could expand on this finding and study the potential of further subpopulations to grow as organoids.

4- Although data presented in figure 1, and supplementary movie, appears to suggest clonal origin for the TEC-derived organoids, have the authors conducted similar experiments using limiting dilution of TEC numbers or single-cell sorting? Alternatively, mixed TECs from reporter mice with distinct fluorescence markers (e.g., RFP vs YFP) could confirm clonality? In this latter system, clonal organoids should be monocromatic, providing more solid evidence of their clonal origin.

We took the different approaches suggested by the reviewer to provide more evidence about the clonal origin of TEC-derived organoids. As also observed for other cell types, a minimum cell density is required for proper organoid formation. The seeding density had previously been optimized for TEC-derived organoid growth and also to avoid overcrowding. Limiting dilution experiments thus did not provide very useful information, as at dilutions where cells can easily be tracked, organoid growth was suboptimal and therefore not representative. We also aimed at sorting single TECs into 96w plate. We could observe about 1% efficiency in the formation of organoid, although they remained very small likely again due to the absence signals from other cells (Figure 1 for reviewers). We did not have access to TECs from reporter mice, but performed similar experiments as suggested by the reviewer using CellTrace dyes. TECs were labelled with two different colors and mixed in a 1:1 ratio before seeding. Although the decrease of the dye intensity with cell divisions (while debris remain extremely bright) renders imaging challenging, we could still observe that organoids were single-colored (Fig. S1C, Movie 2), confirming their clonal origin.

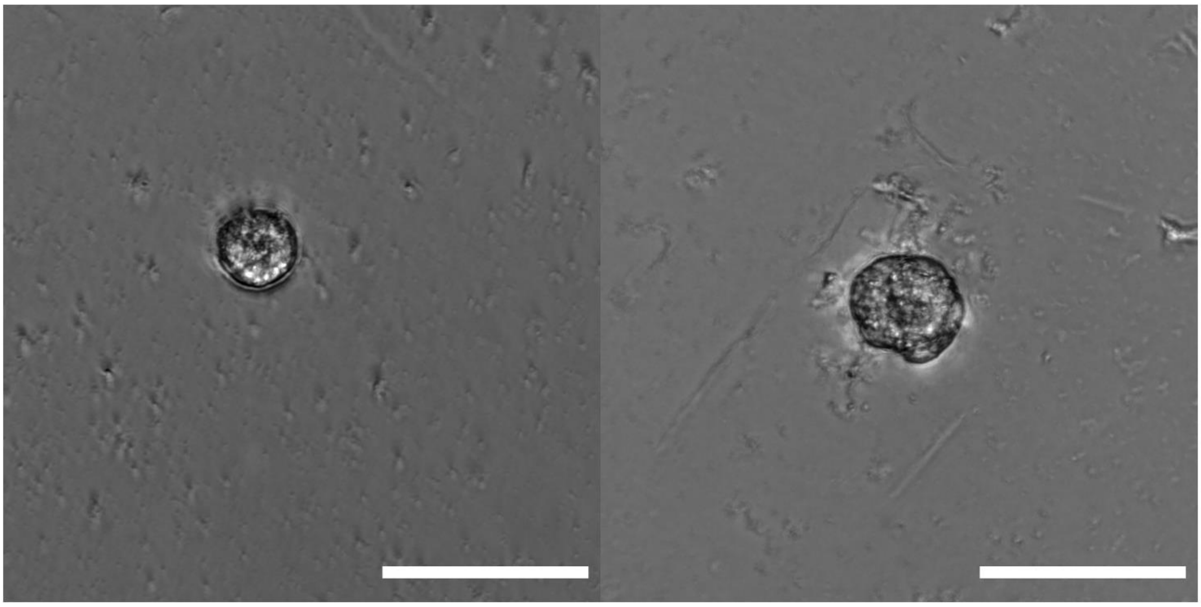

**Figure 1 Organoids from single-cell sorting.** Representative images of two organoids 14 days after single-cell sorting in 96w plate. Scale bars: 100µm.

5 – The genome-wide RNA sequence analysis depicted in Fig 1G seems selective and could be extended to include a larger-scale comparison of differentially expressed genes (DEGs) among ex vivo, organoid, or 2D-derived TECs. This expanded analysis should also include a comprehensive comparison of Foxn1 targets (PMID: 27548434). Specifically, do organoid-derived TECs match ex vivo isolated TECs from E16.5 thymus in terms of the full breadth of Foxn1 targets? This more broad comparison would provide valuable insights into the fidelity of organoid-derived TECs to mimic the transcriptional profile of their ex vivo counterparts.

© 2024. Published by The Company of Biologists under the terms of the Creative Commons Attribution License (https://creativecommons.org/licenses/by/4.0/).

We thank the reviewer for pointing this out. The revised version of the paper includes a supplementary table of genes differentially expressed between conditions. We also compared *Foxn1* targets identified in Žuklys et al. 2016 (Fig. S1H) and showed that the expression levels of genes with the highest expression in freshly extracted TECs were relatively well maintained in organoids and that this was not necessarily the case for 2D-cultured TECs. The filtering criteria applied in our analysis (exclusion of lowly-expressed genes (< 1 counts per million) and of genes present in less than three samples) removed some of the *Foxn1* target genes. We omitted these filtering criteria to show the 30 top Foxn1 targets from Žuklys et al. 2016 in Figure 2 for reviewers.
We also realized that the conclusion sentence of this part of the manuscript was somehow misleading. We wanted to point out that organoids were closer to in vivo TECs than to 2D-cultured TECs, thus making us question whether they could remain functional, and not that organoids were similar to in vivo TECs. We apologize for the confusion and corrected the text accordingly.

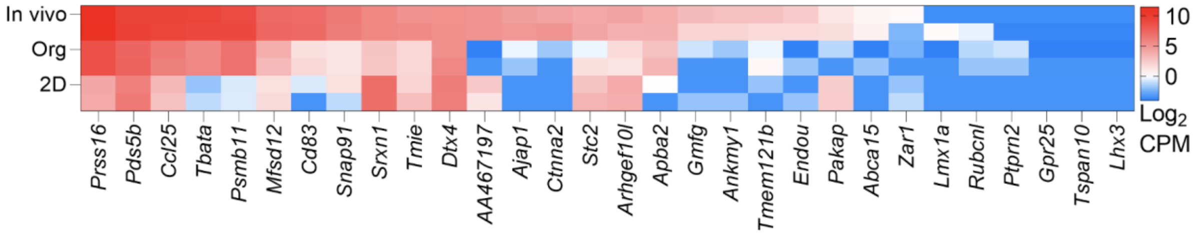


**Figure 2 *Foxn1* target genes expression.** Heatmap displaying the expression of the top 30 *Foxn1* targets identified in Zuklys et al. 2016 in freshly isolated TECs (In vivo), in thymic epithelial organoids and in TECs cultured in 2D (2D). Here no filtering criteria on expression level in a minimal number of samples were applied (in contrary to the main analysis in the paper and Fig. S1H)

6 – The lineage and functional potential assays set in Figures 2-4 appears to be conducted with a pool of TEC-derived organoids. In this system, it is possible that only a fraction of organoids contribute to TEC lineage and/or may contribute to different TEC lineages, such as cortical and medullary subsets. Despite possible phenotypic similarities as shown in Figure 1, possible inter-clonal heterogeneity may determine different lineage and functional potentials. Can the authors comment (and discuss) on this aspect?
We thank the reviewer for this comment. We believe it is similar to point number 7, and therefore addressed it there.

7- The lineage and functional potential assays depicted in Figures 2-4 seem to have been conducted using a pool of TEC-derived organoids. In this setup, it is plausible that only a subset of organoids contributes to the TEC lineage and/or may contribute to different TEC lineages, such as cortical and medullary subsets. Despite potential phenotypic similarities as shown in Figure 1, the presence of inter-clonal heterogeneity could influence distinct lineage and functional potentials. Commenting and discussing this aspect may provide a more balanced understanding of the results and their implications. Additionally, discussing strategies to mitigate possible heterogeneity in future experiments could strengthen the study's conclusions.
These are very pertinent remarks and we thank the reviewer for pointing this out. The cell number needed to generate reaggregates fetal thymic organ cultures (RFTOC) indeed required pooling thymic epithelial organoids. We now highlight this aspect in the discussion part of the revised manuscript and comment on the fact that we cannot exclude the impact of clonal heterogeneity on the functional ability of TECs to mediate T cell development in ORFTOCs. We show that both sorted cTECs and mTECs have the potential to form organoids in point number 3 and Fig. S1E. Future experiments could therefore only use one of the cell types and test their potential to mediate T cell maturation.

Minor point:
Sentence on lines 53-55 might need some revision. It seems to lack clarity or completeness after "which involve organotypic three-dimensional (3D) cultures containing different cell types."
For more clarity, we modified the sentence to *"Finally, while TEC functionality is preserved in (reaggregate) thymic organ culture, these 3D organotypic culture contain different cell types. Therefore, to date no conditions allow the expansion of functional TECs independently, highlighting the need for more advanced culture methods."* in the revised version of the text.

**Reviewer 2** Advance Summary and Potential Significance to Field:

© 2024. Published by The Company of Biologists under the terms of the Creative Commons Attribution License (https://creativecommons.org/licenses/by/4.0/). 

Thymic epithelial cells are a notoriously difficult cell type to successfully culture. With a severely limited number of successful in-vitro thymic organoid systems reported, there is a great need for new methods to successfully support T cell development in culture. With the overall decline in thymic function with age as a consequence of thymic involution, plus many clinical settings where thymic function is either absent or becomes compromised, the need for the development of successful thymic organoid cultures is of high importance, both clinically and to better understand the fundamentals of thymus biology. Hubscher et al aim to conquer the long-standing challenge of generating functional thymic organ cultures, by applying their expert knowledge of organoid systems used for other endoderm-derived organs. Authors clearly demonstrate they have developed a novel thymic organoid protocol, creating 3D thymic structures from embryonic TEC that are able to support the major stages of thymocyte development when reaggregated with T cell precursors. Moreover, when transplanted under the kidney of wildtype mice, the thymic organoids support normal T cell development of host derived T cell precursors. Overall, demonstrating the successful generation of a novel thymic organoid system.
We really appreciate the supportive comments from the reviewer.

**Reviewer 2** Comments for the Author:
1- The manuscript is well written and easy to follow, and the data represents well designed and executed experiments. However, before the acceptance for publication in the Journal of Development, I recommend a number of changes to the manuscript. My most serious concerns are the absence of references to already established thymic organoid systems and the lack of clarity on how the culturing of E16.5 TEC first in the thymic organoid and then reaggregating in ORFTOCs offers an advantage over isolating TEC E16.5 from FTOC (with or without deoxyguanosine treatment) and then using these TEC in the pre-existing RTOC system.
We thank the reviewer for his/her/their comment. In the revised version of the manuscript, we expanded the introduction and discussion to include references to and comments on already established systems to study TECs and T cell development vitro.
Although there are several papers reporting on thymus organoids, the term “organoid” and its definition have unfortunately been inconsistently used. Previously published thymic organoid systems have not been included in recent landmark organoid reviews[1,2,3,4] and would rather be considered as ‘organotypic cultures’ by the organoid field. In contrast, as we focused on developing organoids derived solely from thymic epithelial cells, our thymic epithelial organoids more closely resemble canonical examples of organoids such as those derived from the intestine[5]. Culturing thymic epithelial cells remains extremely challenging[6], and no other method (e.g. 2D) allows to grow them independently of other cell types in a way that preserves their functionality (i.e. ability to mediate T cell maturation).
In this study, we used reaggregate thymic organ cultures primarily to demonstrate that thymic epithelial cells grown as organoids have the potential to mediate T cell maturation and selection when later aggregated with early T cell progenitors. We added a couple of sentences in the discussion to explain the potential advantages of culturing thymic epithelial cells as organoids to study the influence of specific (rare) TEC subtypes on T cell development upon reaggregation with T cell progenitors. The possibility to culture TECs independently of other cell types presents an advantage compared to culturing them as a bulk population and with other cells types in FTOCs before forming RTOCs.

There are additional changes I believe need to be made to the manuscript before it should be considered for publication.

---

[1] Rossi, G., Manfrin, A. & Lutolf, M.P. Progress and potential in organoid research. Nat Rev Genet 19, 671–687 (2018). https://doi.org/10.1038/s41576-018-0051-9
[2] Hofer, M., Lutolf, M.P. Engineering organoids. Nat Rev Mater 6, 402–420 (2021). https://doi.org/10.1038/s41578-021-00279-y
[3] Zhao, Z., Chen, X., Dowbaj, A.M. et al. Organoids. Nat Rev Methods Primers 2, 94 (2022). https://doi.org/10.1038/s43586-022-00174-y
[4] Kim, J., Koo, BK. & Knoblich, J.A. Human organoids: model systems for human biology and medicine. Nat Rev Mol Cell Biol 21, 571–584 (2020). https://doi.org/10.1038/s41580-020-0259-3
[5] Sato, T., Vries, R. G., Snippert, H. J., et al. Single Lgr5 stem cells build crypt-villus structures in vitro without a mesenchymal niche. Nature, 459(7244), 262-265 (2009). https://doi.org/10.1038/nature07935
[6] Michaels, Y.S., Buchanan, C.F., Gjorevski, N. et al. Bioengineering translational models of lymphoid tissues. Nat Rev Bioeng 1, 731–748 (2023). https://doi.org/10.1038/s44222-023-00101-0

© 2024. Published by The Company of Biologists under the terms of the Creative Commons Attribution License (https://creativecommons.org/licenses/by/4.0/).

2- The introduction is too short and brief. There is no clear explanation of what has previously been published in the thymic organoid field, nor has any explanation been given to the importance or clinical significance of successfully replicating T cell development in a thymic organoid system. A major expansion of the introduction is essential to highlight the novelty and impact of the work presented. Moreover, the abstract would also be strengthened if amended to include these changes. More justification is also needed to strengthen the logic of why applying approaches used for other endoderm-derived organs would improve pre-existing thymic culture systems. Additionally, no description is provided of how this approach is different compared to non-endoderm-derived organoids.
We thank the reviewer his/her/their suggestion on how to improve the introduction and abstract. We implemented the requested changes to include previous publications on methods to grow thymic epithelial cells and to study T cell development in vitro. We also mentioned the potential clinical relevance of the work once translated to human TECs.
Thymic epithelial cells are notoriously difficult to culture as they tend to lose their functional ability to mediate T cell maturation. Given the fact that organoids have been proven more physiologically relevant that other culture methods, we proposed to apply this approach to grow TECs. Most bona fide organoids (i.e. derived from single stem/progenitor cells such as the canonical example of the intestine) model tissues originating from the endodermal germ layer (reviewed in Rossi et al. 2018)[7]. Given the endodermal origin of TECs, we thought that this approach could be suitable as well to generate thymic epithelial organoids.

3- No justification is provided for why embryonic day E16.5 has been chosen. Could this method be applied to younger and older TEC sources. Would it work using NB and adult TEC? If so, this would clearly highlight the advantage of the thymic organoid system over traditional FTOC to RTOC methods. Moreover, authors suggest this organoid system may be able to maintain long term functionality and data is presented up to 13 days in culture. Can this culture system remain functional for over 13 days, highlighting an additional advantage of the thymic organoid system over traditional FTOC to RTOC methods.
We thank the reviewer for this question. E16.5 thymi have initially been chosen for practical reasons. At E16.5, thymi contain a sufficient number of TECs to efficiently sort them and to generate one batch of organoids using embryos originating from one pregnant mother. At younger age, embryos originating from several pregnant females would have to be pooled. This increases significantly the number of mice required for experiments. In addition, the TEC/thymocyte ratio in E16.5 thymi is still ideal and cells do not necessitate EpCAM-enrichment or CD45-depletion before sorting. This extra step is often needed when using older thymi[8] and results in longer procedures and cell loss.
In the current culture conditions, organoids can also be generated from neonatal (P0) sorted TECs. This data has been added to the updated version of the manuscript (Fig. S2F).
Thymic epithelial organoids are usually used at D7 for reaggregation experiments. This is because maintaining their functionality over time remains challenging in the current culture conditions, as discussed in the text. Regarding ORFTOCs, we included data from D21 cultures in the revised version of the manuscript (Fig. 2F, Fig. S2J and N).

4- Mouse embryonic fibroblasts (MEFs) were added to the reaggregates. How essential is this step. Would the organoids work without the addition of MEFs? The inclusion of a without MEF control group would address this issue.
Initially we added MEFs to the reaggregates because we followed the method described in Sheridan et al. 2009[9]. MEFs increase pellet size and therefore facilitate handling. We agree with the reviewer suggestion to include a control group without MEFs. This new data has been added to the revised version of the manuscript (Fig S2E), and no major differences compared to reaggregates comprising MEFs were observed.

---

[7] Rossi, G., Manfrin, A. & Lutolf, M.P. Progress and potential in organoid research. Nat Rev Genet 19, 671–687 (2018). https://doi.org/10.1038/s41576-018-0051-9
[8] Xing, Y., & Hogquist, K. A. Isolation, identification, and purification of murine thymic epithelial cells. JoVE (Journal of Visualized Experiments), (90), e51780 (2014). doi: 10.3791/51780
[9] Sheridan, J. M., Taoudi, S., Medvinsky, A., & Blackburn, C. C. A novel method for the generation of reaggregated organotypic cultures that permits juxtaposition of defined cell populations. Genesis, 47(5), 346-351 (2009). https://doi.org/10.1002/dvg.20505

© 2024. Published by The Company of Biologists under the terms of the Creative Commons Attribution License (https://creativecommons.org/licenses/by/4.0/).

5- The findings of the manuscript would be strengthened by the extension of the bulk RNA seq data analysis displayed in Fig. 1J. Is EpCAM expressed? What is the expression of mTEC markers such as CCL21a and Aire? Do you see TRA expression? Aire dependent and Aire independent TRA?
We thank the reviewer for this interesting comment. *Epcam*, *Ccl21a* and *Tnfrsf11a* expression were added to the heatmap in Fig. 1J. The filtering criteria applied in the analysis (exclusion of lowly-expressed genes (< 1 counts per million) and of genes present in less than three samples) however did not permit to detect *Aire* expression. We nonetheless provide here (Figure 3 for reviewers) a heatmap showing the expression of *Aire* and of several TRAs without applying filtering criteria. It seems that most of them are very lowly expressed, even in freshly isolated E16.5 TECs. *Aire* however is present in freshly isolated cells and mostly lost in organoids. We can speculate that mature mTECs expressing *Aire* die over the culture period and/or that the culture conditions favor more progenitor-like TEC phenotypes. The absence of crosstalk might prevent the maturation of new *Aire*-positive mTECs in the organoid culture. However, upon reaggregation with T cell progenitors, *Aire*-expressing cells can been detected, as shown in the single-cell sequencing data (Fig. S3B).
We also realized that the conclusion sentence of the bulk RNA sequencing part was somehow misleading. We wanted to point out that organoids were closer to in vivo TECs than to 2D-cultured TECs, thus making us question whether they could remain functional, and not that organoids were similar to in vivo TECs. We apologize for the confusion and corrected the manuscript accordingly.

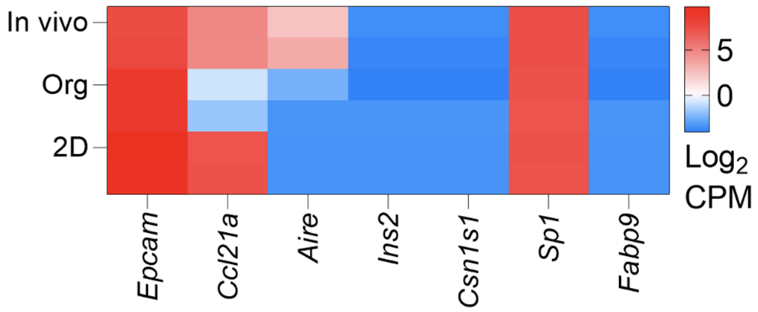


**Figure 3 Expression of *EpCAM* and mTECs genes.** Heatmap representing genes expression without filtering criteria in freshly isolated E16.5 TECs (In vivo), in thymic epithelial organoids (Org) and in TECs culture in 2D (2D).

6- The direct and indirect effects of FGF7 are unclear. Does the increase proliferation drive the changes in metabolism or vice versa. This relationship could be discussed in the text.
We now state in the discussion that previous studies have shown that FGF7 binding to FGFR2b leads to signaling through the Ras/MAPK and PI3K/Akt/mTOR cascades[10]. These pathways regulating proliferation and metabolism, respectively, both seem to be direct targets of FGF7. We did not study further the signaling cascade of FGF7 in thymic epithelial organoids and this could be the scope of a follow-up paper.

7- What are the numbers obtained from organoids? Can counts be provided? What numbers of TEC are put into the ORFTOCs and do they increase in size over the culture period? Is this at a comparable rate as the FTOC controls?
We thank the reviewer for these questions.
We now included a graph displaying numbers for the main thymocyte populations in ORFTOCs analyzed by flow cytometry (Fig. S2L).
100 000 organoid-derived TECs were used to generate ORFTOCs. Reaggregates do increase in size during the culture period. Precise size and volume quantification over time is however difficult to perform given the culture method on membrane filters, which is not very compatible with imaging (filters are not transparent for imaging from below, and similar in color as the reaggregates when imaged from above). Figure 4 for reviewers illustrates the evolution of representative ORFTOC and

[10] Rossi, Simona W., et al. "Keratinocyte growth factor (KGF) enhances postnatal T-cell development via enhancements in proliferation and function of thymic epithelial cells." Blood 109.9, 3803-3811 (2007). https://doi.org/10.1182/blood-2006-10-049767

© 2024. Published by The Company of Biologists under the terms of the Creative Commons Attribution License (https://creativecommons.org/licenses/by/4.0/). 

E13.5 FTOC from the day after seeding to two weeks later. For ORFTOC at D1, only the outline of a drop can be observed. Over time, the cells form a small aggregate that increases in size.

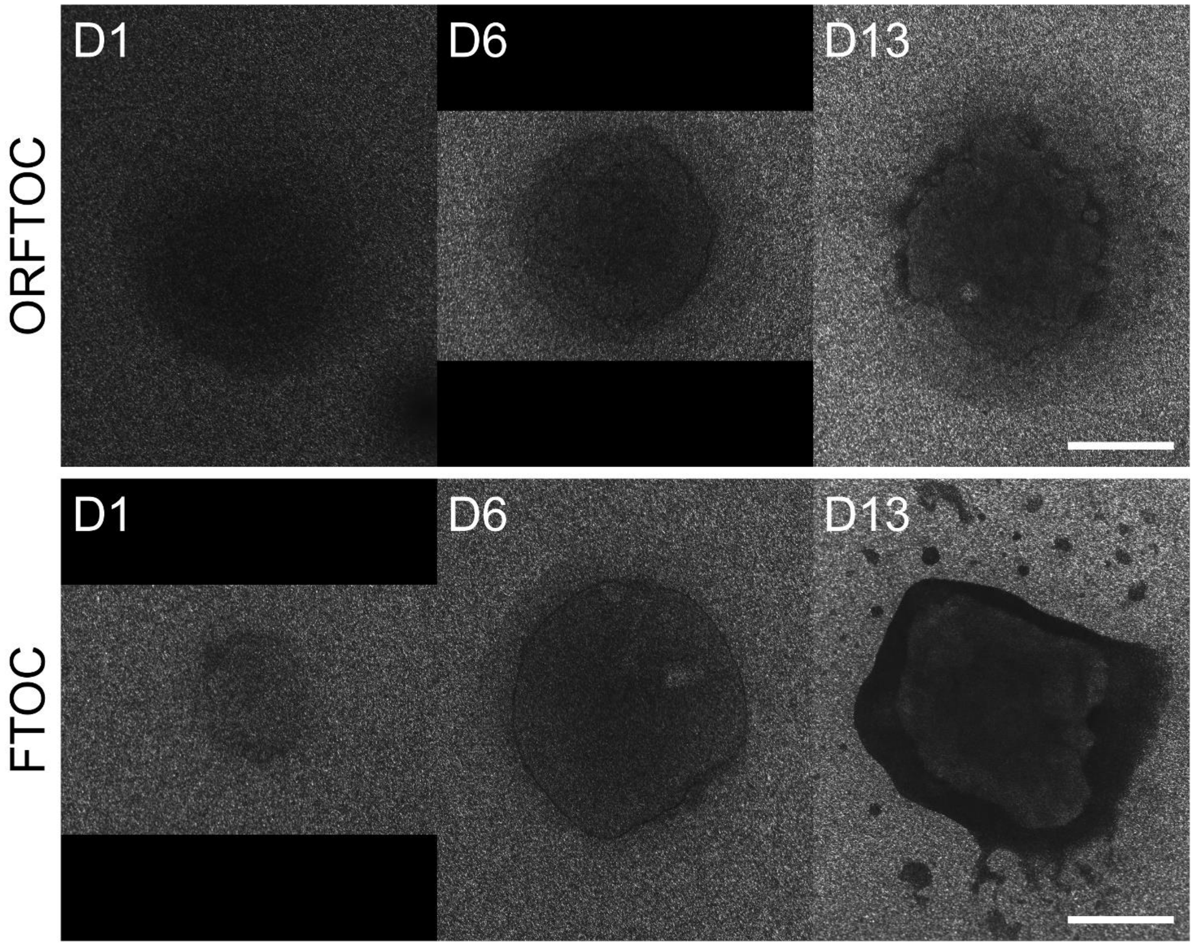


**Figure 4 ORFTOC and FTOC evolution over time.** Representative images (from below, through the membrane) of ORFTOC and E13.5 FTOC at D1, D6 and D13. Scale bars: 1000µm.

At end points, ORFTOC and FTOC can be detached from the membrane filter and imaged with a stereomicroscope (Figure 5 for reviewers). Although the reaggregates are a bit rounder, more irregular and smaller than entire lobes, no striking difference can be observed by eye.

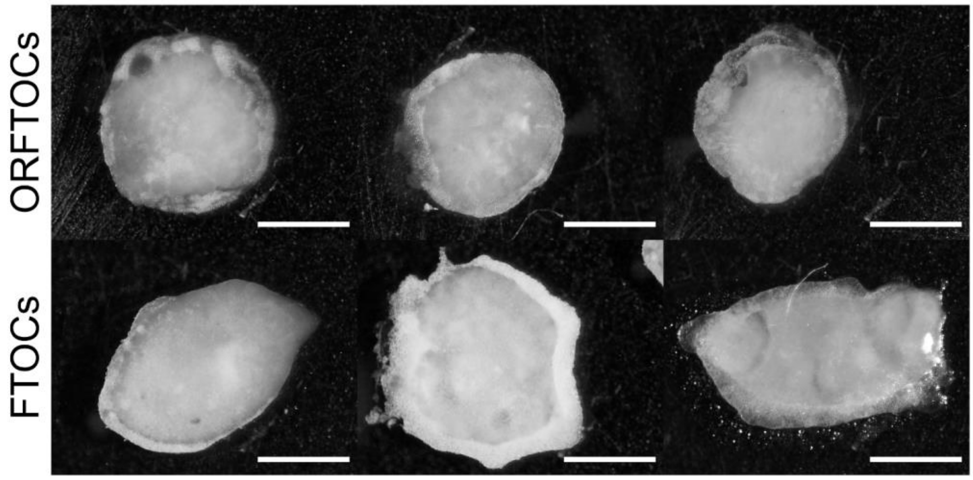


**Figure 5 ORFTOC and FTOC comparison.** After 14 days in culture, ORFTOCs and E13.5 FTOCs were detached from the membrane and imaged. Scale bars: 1000µm.

© 2024. Published by The Company of Biologists under the terms of the Creative Commons Attribution License (https://creativecommons.org/licenses/by/4.0/).

8- It is unclear if authors can disaggregate the ORFTOCs and stained for TEC markers by flow cytometry post culture, or this can only be done after grafting (Fig. S4). Moreover, in Figure 3D it is unclear how many ORFTOC were sorted for the scRNA and if several ORFTOC were pooled?
As Fig. S4 shows thymocyte staining, we are unsure about the first part of this reviewer question. Although we did not perform this routinely because our analysis panel mostly focused on thymocytes, we demonstrate in Figure 6 for reviewers that ORFTOCs can be disaggregated and stained for TEC markers such as CD205 and UEA1 by flow cytometry post culture. Freshly extracted E16.5 thymi were used for setting gates.

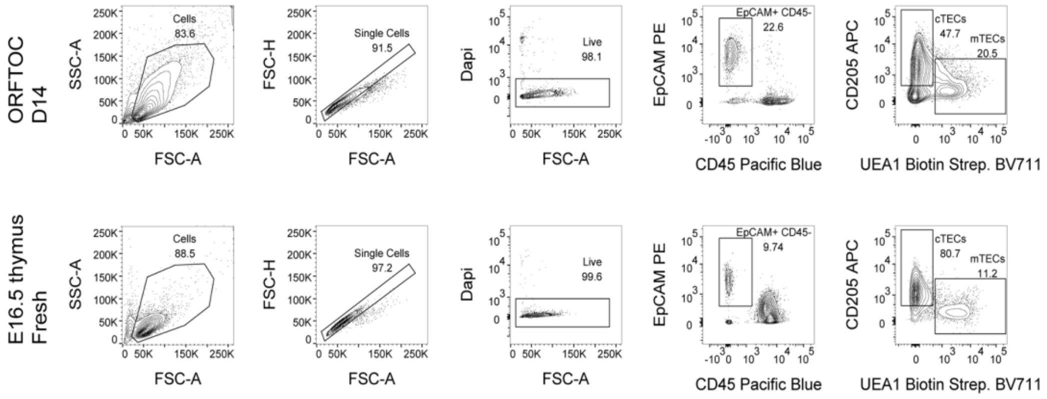


**Figure 6 TEC marker analysis by flow cytometry.** After 14 days in culture, ORFTOCs were dissociated, stained for the TEC markers CD205 (cTECs) and UEA1 (mTECs) and analyzed by flow cytometry. A representative example is shown here. Gates were set based on cells freshly isolated from E16.5 thymi.

For scRNAseq analysis, two ORFTOCs were pooled as explained in the methods. We now included this information in the figure legend.

9- FTOC controls for scRNAseq were E13.5, but the TEC from ORFTOCs originated from E16.5 in the thymic organoids, therefore could this explain some of the differences observed. It also needs to be made clearer the age of the reference populations from Park et al. reference atlas (Fig. 3, E and F).
We agree with the reviewer observation that embryonic age used differs between TEC in ORFTOCs and TECs in FTOC controls. As stated in the manuscript, we initially aimed at matching the embryonic age of the T cell progenitors to be able to compare T cell development. Moreover, we started with E13.5 thymi to have T cell progenitors at the early DN stages. On the other hand, thymic epithelial organoids were initially generated from E16.5 thymi for practical reasons, as explained in point number 3.
The age of the reference populations from the Park et al. reference atlas are now clearly indicated in the methods and in the figure legend. Reference TECs span from E14.5 to 4-6 week-old. For T cells, we used the youngest adult dataset (4-week-old) generated in their study. We based that decision on the fact that the prenatal data does not contain thymocytes past the DP proliferative stage and does not fully align with the postnatal data even in the original publication (Figure 7 for reviewers).

NOTE: Figure provided for reviewer has been removed. It showed Figure S20 from **Park, J. E., Botting, R. A., Domínguez Conde, C., Popescu, D. M., Lavaert, M., Kunz, D. J., Goh, I., Stephenson, E., Ragazzini, R., Tuck, E., et al.** (2020). A cell atlas of human thymic development defines T cell repertoire formation. *Science* **367**, eaay3224. doi:10.1126/science.aay3224. We have removed unpublished data that had been provided for the referees in confidence.

10- ORFTOC were transplanted under the kidney capsule of syngeneic CD45.1 recipient mice and harvest 5 weeks later. The data demonstrated the grafts were able to support the major stages of T cell development (DN/DP/SP4/SP8 subsets, and Treg). Were a population of CD45.1+ ORFTOC

© 2024. Published by The Company of Biologists under the terms of the Creative Commons Attribution License (https://creativecommons.org/licenses/by/4.0/). 

derived T cells detectable in the periphery of the recipients/hosts? This would suggest the T cells made in vitro were functional and could contribute to the peripheral pool of T cells.

We thank the reviewer for this very interesting question. We unfortunately could not detect a population of CD45.2 ORFTOC-derived T cells in the periphery of the graft recipient. We believe that this is due to a dilution effect, as T cell originating from the graft are competing with those from the endogenous thymus. We are now transparent about this and have included this consideration in the discussion. Of note, we could not detect CD45.2 FTOC derived T cells in FTOC recipient mice either. Grafting in nude mice would most likely be required to be able to detect donor T cells in the periphery. We however do not have an animal license allowing such experiments.

Alternatively, we aimed at testing the functionality of ORFTOC-derived T cells by activating them with CD3/CD28 beads. We cultured ORFTOCs for 17 days, dissociated them, separated the cells equally into two wells, and added beads (Dynabeads® Mouse T-Activator CD3/CD28, Cat. no. 114.56D) to one of them. After 3 days, cells were analyzed by flow cytometry and we could observe a significantly different proportion and number of SP4 and SP8 T cells expressing the activation markers CD44 and/or CD25 in the condition with beads compared to controls (Figure 8 for reviewers).

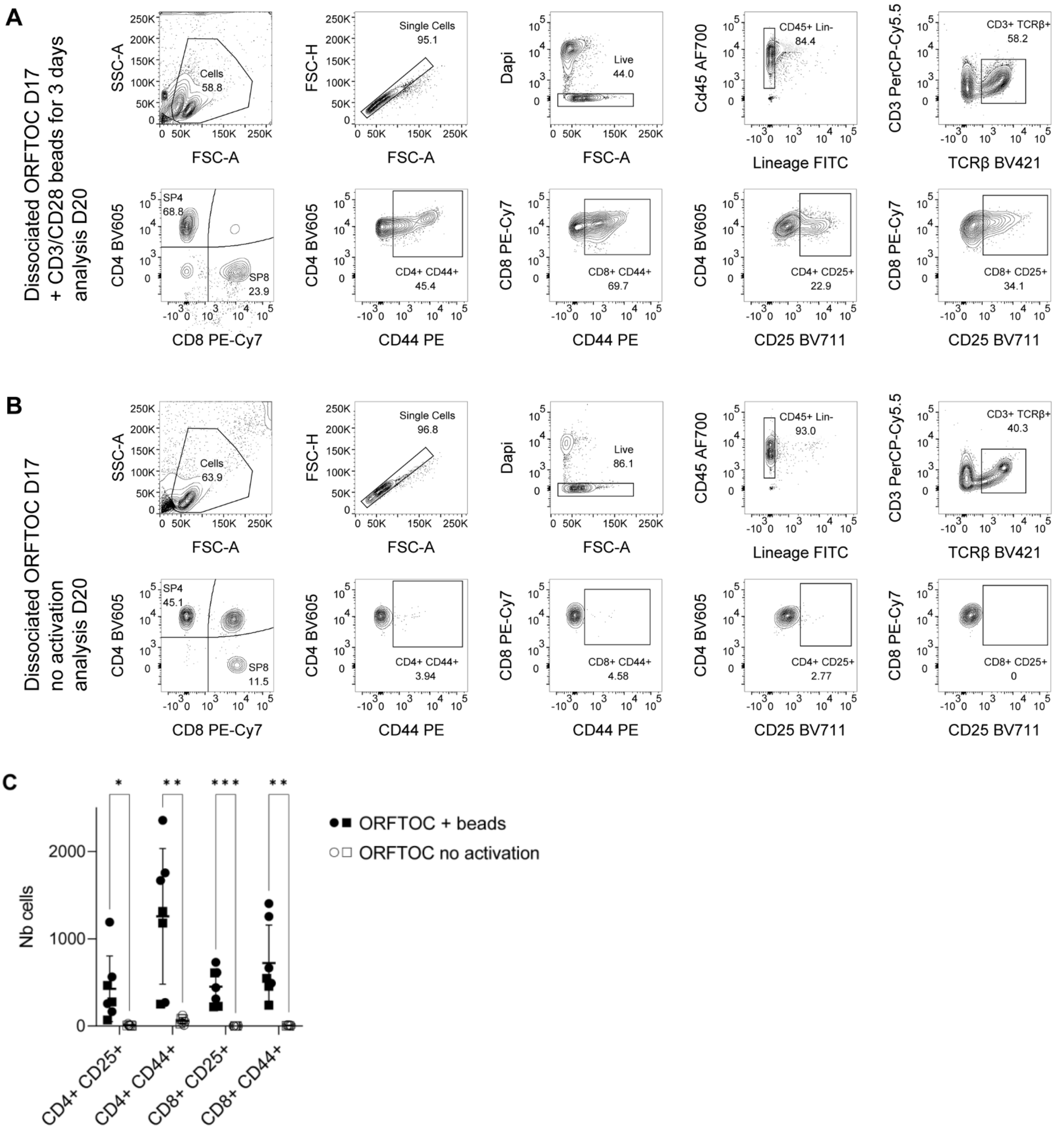


© 2024. Published by The Company of Biologists under the terms of the Creative Commons Attribution License (https://creativecommons.org/licenses/by/4.0/). 

**Figure 8 T cell activation with CD3/CD28 beads.** (**A-B**) D17 ORFTOC were dissociated, equally distributed in two wells and cultured for 3 days in 96w plate in the presence (A) or in the absence (B) of CD3/CD28 beads before being analyzed by flow cytometry. (**C**) From flow cytometry analysis, quantification of the activated populations. *: P = 0.012633, **: P = 0.001547 (CD4+ CD44+), **: P = 0.0009535 (CD8+ CD44+), ***: P = 0.000088 (multiple unpaired t-tests); n = 7 ORFTOC from two independent experiments (circle or square represent the different experiments).

11- Representative immnostaining confirming medullary and cortical regions are not very clear in figure 4G. Can images of a whole section of the graft be displayed that allows the overall assessment of proportion of medullary to cortical areas? TEC markers could be shown, but also CD4 and CD8 staining would reveal DP enriched cortical regions vs SP4/SP8 enriched medullary regions.
Following the reviewer suggestion, we added a whole section immunostaining showing UEA1 and KRT8 in grafted ORFOTC and in grafted FTOC for comparison in the revised version of the manuscript (Fig. S4H,I).

12- Are the T cells that are generated in the ORFTOC tolerant? Have the ORFTOC been grafted under the kidney capsule of nude mice and if so, do hosts show signs of autoimmunity? This would be an insightful experiment but is not essential for publication.
This is a very insightful comment that we thank the reviewer for bringing up. We however did not perform this experiment as we believed it was rather the scope of a follow-up paper. We included a comment in the discussion on how grafting ORFTOC in nude mice might provide insights on T cell tolerance and ability to mount an immune response.

---

Second decision letter

MS ID#: DEVELOP/2024/202853

MS TITLE: Thymic epithelial organoids mediate T cell development

AUTHORS: Tania Hubscher, Francisco Lorenzo-Martin, Thomas Barthlott, Lucie Tillard, Jakob Langer, Paul Rouse, Clare Blackburn, Georg Clare Hollander, and Matthias Lutolf

I am happy to tell you that your manuscript has been accepted for publication in Development, pending our standard publication integrity checks.

Reviewer 1

*Advance summary and potential significance to field*

The authors have done a thorough revision of the manuscript according to my comments.

*Comments for the author*

The addition of new data and changes to the text have improved the scope of the study.

Reviewer 2

*Advance summary and potential significance to field*

Authors have successfully addressed my concerns and amended the manuscript accordingly. I fully endorse the publication of this manuscript in Development.

*Comments for the author*

© 2024. Published by The Company of Biologists under the terms of the Creative Commons Attribution License (https://creativecommons.org/licenses/by/4.0/).

Authors have successfully addressed my concerns and amended the manuscript accordingly. I fully endorse the publication of this manuscript in Development.

© 2024. Published by The Company of Biologists under the terms of the Creative Commons Attribution License (https://creativecommons.org/licenses/by/4.0/).
